# Does Spike-Timing-Dependent Synaptic Plasticity Couple or Decouple Neurons Firing in Synchrony?

**DOI:** 10.3389/fncom.2012.00055

**Published:** 2012-08-21

**Authors:** Andreas Knoblauch, Florian Hauser, Marc-Oliver Gewaltig, Edgar Körner, Günther Palm

**Affiliations:** ^1^Honda Research Institute EuropeOffenbach/Main, Germany; ^2^Institute of Neural Information ProcessingUlm University, Ulm, Germany; ^3^The Blue Brain Project, Ecole Polytechnique Federale de LausanneLausanne, Switzerland

**Keywords:** Hebbian cell assemblies, learning, memory, spike synchronization, STDP, synaptic connectivity, synaptic plasticity

## Abstract

Spike synchronization is thought to have a constructive role for feature integration, attention, associative learning, and the formation of bidirectionally connected Hebbian cell assemblies. By contrast, theoretical studies on spike-timing-dependent plasticity (STDP) report an inherently decoupling influence of spike synchronization on synaptic connections of coactivated neurons. For example, bidirectional synaptic connections as found in cortical areas could be reproduced only by assuming realistic models of STDP and rate coding. We resolve this conflict by theoretical analysis and simulation of various simple and realistic STDP models that provide a more complete characterization of conditions when STDP leads to either coupling or decoupling of neurons firing in synchrony. In particular, we show that STDP consistently couples synchronized neurons if key model parameters are matched to physiological data: First, synaptic potentiation must be significantly stronger than synaptic depression for small (positive or negative) time lags between presynaptic and postsynaptic spikes. Second, spike synchronization must be sufficiently imprecise, for example, within a time window of 5–10 ms instead of 1 ms. Third, axonal propagation delays should not be much larger than dendritic delays. Under these assumptions synchronized neurons will be strongly coupled leading to a dominance of bidirectional synaptic connections even for simple STDP models and low mean firing rates at the level of spontaneous activity.

## 1 Introduction

Whether neural activity follows either a rate code or a temporal code (Singer and Gray, 1995; Theunissen and Miller, 1995; Shadlen and Movshon, 1999; VanRullen et al., 2005; Clopath et al., 2010) and, in the latter case, whether spike synchronization will either couple or decouple coactivated neurons (Lubenov and Siapas, 2008; Clopath et al., 2010; Fell and Axmacher, 2011) are still unsolved issues in neuroscience. These questions bear importance for both understanding brain functions and improving therapy of diseases such as epilepsy, tinnitus, and Parkinson (Lubenov and Siapas, 2008; Benabid et al., 2009; Pfister and Tass, 2010).

On the one hand, there is physiological evidence that spike synchronization reflects feature integration (Singer and Gray, 1995), attention (Fries et al., 2001), and associative learning (Miltner et al., 1999), which suggests a constructive rather than destructive role of spike synchronization for memory (Jutras and Buffalo, 2010; Fell and Axmacher, 2011) and the formation of Hebbian cell assemblies (Hebb, 1949; Braitenberg, 1978; Palm, 1982; Knoblauch and Palm, 2002a; Pulvermüller, 2003; Lansner, 2009; Buzsaki, 2010). Consistent with many attractor neural network models of memory (Marr, 1971; Palm, 1980; Hopfield, 1982; Lansner, 2009; Knoblauch, 2011), these ideas imply the prediction that synchronized neurons should organize into bidirectionally connected cell ensembles, where the presence of a strong synapse from neuron *i* to neuron *j* increases the likelihood for the presence of a strong synapse in the reverse direction from neuron *j* to neuron *i*, as has been reported for various cortical areas (Markram et al., 1997a; Song et al., 2005).

**Figure 1:**
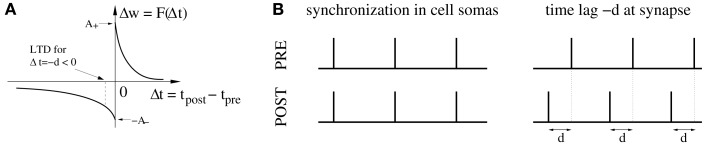
**The conflict between spike-timing-dependent plasticity (STDP) and spike synchronization in Hebbian cell assemblies**. **(A)** In standard spike pair-based (doublet) STDP models, the weight change Δ*w* is a function *F*(*Δ*t) of the time lag Δ*t*: = *t*_post_ − t_pre_ between presynaptic and postsynaptic spike. For positive time lags Δ*t* > 0 (pre-before-post) STDP models predict long-term potentiation (LTP) with Δ*w* > 0. For negative time lags Δ*t* < 0 (post-before-pre) STDP models predict long-term depression (LTD) with Δ*w* < 0. (**B)** Experimental evidence suggests that spike synchronization reflects constructive processes such as feature integration (Singer and Gray, 1995), attention (Fries et al., 2001), associative learning (Miltner et al., 1999), and memory formation (Jutras and Buffalo, 2010; Fell and Axmacher, 2011) which is thought to involve coupling of synchronized neurons into bidirectionally connected Hebbian cell assemblies (Hebb, 1949; Marr, 1971; Braitenberg, 1978; Palm, 1980, 1982; Hopfield, 1982; Knoblauch and Palm, 2002a; Pulvermüller, 2003; Lansner, 2009; Buzsaki, 2010; Knoblauch, 2011). However, these ideas are in conflict with STDP models because, assuming positive axonal propagation delays *d*, synchronization of spike activity in cell somas (left panel) corresponds to negative time lags Δ*t* = −*d* at the synaptic site [right panel; cf. **(A)**]. Therefore, STDP models predict decoupling of neurons firing in synchrony (Gerstner et al., 1996; Song and Abbott, 2001; Knoblauch and Sommer, 2003; Kozloski and Cecchi, 2008; Lubenov and Siapas, 2008; Clopath et al., 2010). It is therefore an open question how unequivocal LTP is accomplished by spike synchronization (Fell and Axmacher, 2011). Here we argue that unequivocal LTP can easily be explained by coarse spike synchronization where time lags are *distributed* around −*d* leading to a mixture of both LTD and LTP events with LTP dominating for plausible model parameters (e.g., LTP amplitude *A*_+_ larger than LTD amplitude *A*_−_; see Discussion; see Figures 6B,D).

On the other hand, it has been pointed out (Fell and Axmacher, 2011) that there is a conflict between these ideas and properties of spike-timing-dependent plasticity (STDP) of synapses (Markram et al., 1997b; Bi and Poo, 1998; Sjöström et al., 2001; Froemke and Dan, 2002). For example, it is well established that the weight modification *F*(Δ*t*) after a spike pairing depends in a characteristic way on the time lag Δ*t*: = *t*_post_ − *t*_pre_ between presynaptic and postsynaptic spike times (Figure 1A). Around Δ*t* = 0 pair-based STDP models assume a sharp transition from maximal long-term depression (LTD) to maximal long-term potentiation (LTP) as the time lags increase from negative to positive values (Gerstner et al., 1996; Song et al., 2000; Izhikevich and Desai, 2003; Morrison et al., 2008), whereas some physiological experiments report a narrow (few milliseconds) transition zone where both LTP and LTD are possible (Bi and Poo, 1998). Even worse, recent theoretical studies on STDP suggest that strong bidirectional synaptic connections would be generally incompatible with temporal coding because model simulations including realistic propagation delays show a strong depression of synapses connecting synchronized neurons (Gerstner et al., 1996; Song and Abbott, 2001; Knoblauch and Sommer, 2003; Kozloski and Cecchi, 2008; Lubenov and Siapas, 2008; Clopath et al., 2010), unless axonal propagation delays are very large (Swadlow, 2000; Knoblauch and Sommer, 2003, 2004), or dendritic delays dominate over axonal delays (Morrison et al., 2007). Intuitively, Hebbian STDP will consistently induce LTD in synapses that connect two neurons firing in synchrony because, at the synaptic site, the presynaptic spike arrives after the postsynaptic spike due to axonal transmission delays (Figure 1B). As pointed out by Fell and Axmacher (2011; Box 2) it is therefore an open question how unequivocal LTP is accomplished by zero-lag phase synchronization.

Still, strong unidirectional connections will result for non-synchronous temporal correlations, for example, if neuron *i* always fires briefly before neuron *j*. Then STDP induces long-term potentiation (LTP) for the connection from neuron *i* to neuron *j*, but LTD for the reverse direction (Markram et al., 1997b; Bi and Poo, 1998; Sjöström et al., 2001; Froemke and Dan, 2002). Together, for synfire-chain-type dynamics (Griffith, 1963; Abeles, 1982; Diesmann et al., 1999) with sequentially activated neuron pools as investigated in the recent paper of Clopath et al. (2010), LTD occurs for within-pool synapses between synchronized neurons, whereas LTP occurs for synapses from one pool to its successor pool. This results in a dominance of unidirectional connections as reported for somatosensory cortex (Lefort et al., 2009; but see Markram et al., 1997a for opposite findings). Explaining the bidirectional connections of visual cortex (Song et al., 2005) has proven more difficult for simple doublet STDP models (Song and Abbott, 2001; Clopath et al., 2010). At least Clopath et al. (2010) have demonstrated stable bidirectional connections for realistic voltage-based STDP models and rate coding where signals are transmitted by neuron pools elevating Poissonian firing rates on a larger time scale of perhaps several hundred milliseconds. It is thus tempting to conclude that unidirectional and bidirectional connections could be signatures of temporal and rate coding, respectively (Clopath et al., 2010).

In this study we question such conclusions by showing that, for realistic model parameters, zero-lag synchronization leads to unequivocal potentiation of synapses connecting coactivated neurons. This answers the question of Fell and Axmacher and reconciles STDP with the ideas described above that neuronal synchronization has an essentially constructive role, for example, for associative learning and memory formation. Specifically, the following shows that STDP consistently couples synchronized neurons if key model parameters are matched to physiological data: First, synaptic potentiation must be significantly stronger than synaptic depression for small (positive or negative) time lags between presynaptic and postsynaptic spikes. Second, spike synchronization must be sufficiently imprecise, for example, within a time window of 5–10 ms instead of 1 ms. Third, axonal propagation delays should not be much larger than dendritic delays. Under these assumptions synchronized neurons will be strongly coupled leading to a dominance of bidirectional synaptic connections even for simple STDP models and low mean firing rates at the level of spontaneous activity. Our conclusions are supported by analyses and simulations of various different STDP models (Morrison et al., 2008; Clopath et al., 2010). Section 2.1 reevaluates prior studies (Lubenov and Siapas, 2008; Clopath et al., 2010) claiming that STDP would generally decouple synchronized neurons and presents modified simulation experiments that put these earlier findings into perspective by demonstrating a strong coupling force of coarse synchronization that can easily explain the dominance of strong bidirectional connections both for sequential and non-sequential temporal codes. Section 2.2 works out the basic mechanism for this effect by analyzing and simulating various STDP models. This includes simple linear doublet models, non-linear doublet models, and the more realistic triplet STDP model (Pfister and Gerstner, 2006; Morrison et al., 2008) fitted to physiological data from visual cortex and hippocampus. Finally, the results are summarized and discussed in Section 3.

## 2 Results

### 2.1 Decoupling through synchrony? a reevaluation of prior studies

We first investigated a realistic voltage-based STDP model proposed by Clopath et al. (2010). This model has been shown to be consistent with a vast literature of physiological experiments on STDP. It has also been used to explain different patterns of synaptic connectivity that seem to occur in different cortical areas (Lefort et al., 2009; but see Markram et al., 1997a) and to relate these connectivity patterns to the underlying neural code. Specifically, they observed strongly unidirectional connections if synaptic inputs were highly structured on the spike time scale according to a synfire-type sequential temporal code, whereas bidirectional connections could be reproduced only for rate coding using stationary stimulation on a larger time scale. For the latter, they emphasized the importance of using their realistic STDP model as commonly used simpler doublet STDP models would be generally unable to stabilize bidirectional connections even under rate coding. In any case, their results suggest that unidirectional and bidirectional connections could be signatures of temporal and rate coding, respectively.

**Figure 2:**
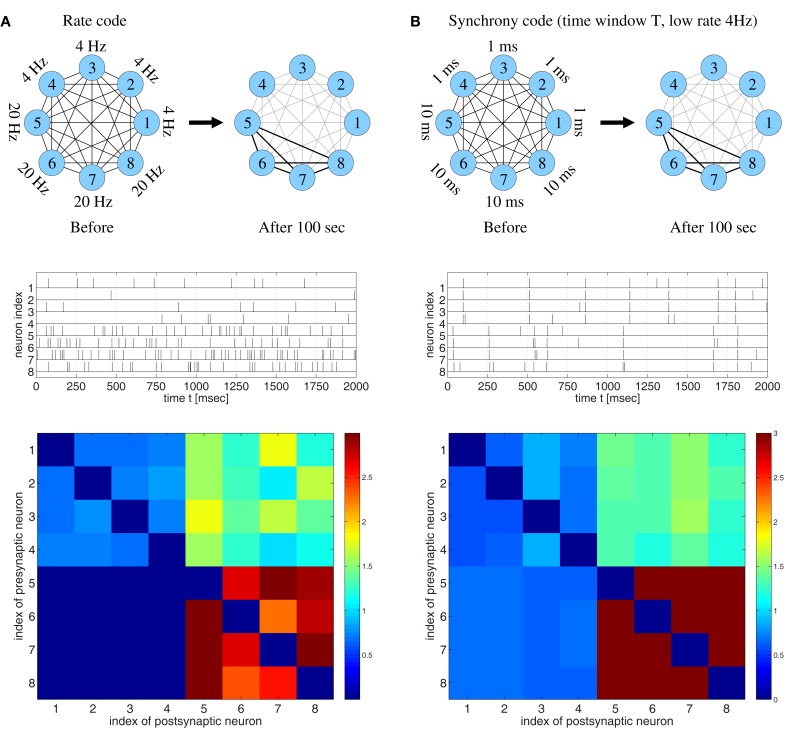
**Strong bidirectional connections can develop both for rate coding and for temporal coding based on spike synchronization assuming realistic STDP models fitted to data from visual cortex (Clopath et al., 2010)**. **(A)** Rate code. Eight neurons fired randomly at different frequencies as indicated (top and middle). Synaptic weights *w_ij_* after 100 s (bottom) indicate that neurons firing at high rates (#5–#8) develop strong bidirectional connections, similarly as reported previously (cf., Clopath et al., 2010, Figure 4). **(B)** Temporal code based on spike synchronization. Eight neurons fired at a low rate (4 Hz) but in synchrony with different synchronization windows *T*. The weights averaged over 100 s indicate that neurons that are coarsely synchronized (*T* = 10 ms; #5–#8) develop strong bidirectional connections in spite of low firing rates. No strong connections develop for either too precise (*T* = 1 ms; #1–#4) or too coarse synchronization [cf. **(A)**]. Synaptic weights were initially *w_ij_*(0) = 1 and clipped throughout the simulations, 0 ≤ *w*_ij_(*t*) ≤ 3. Synaptic delays were *d* = 1 ms.

**Figure 3:**
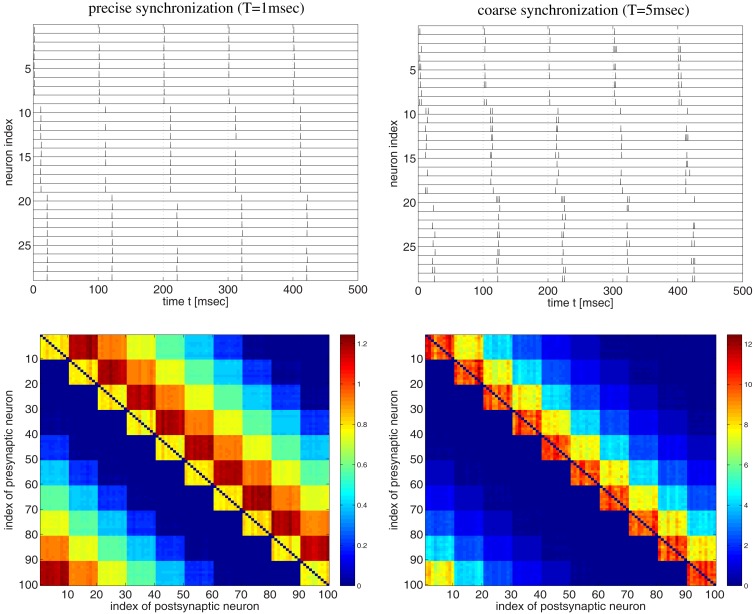
**Strong bidirectional connections can develop also for a synfire-type sequential temporal code**. This figure shows spike recordings (top panels) and final weight matrices (bottom panels, initial weights were 1, no weight clipping) after 100 s simulation of a realistic voltage-based STDP model (Clopath et al., 2010). Simulations are similar as in Figure 2 but here the network comprised 100 neurons divided into 10 pools of 10 neurons (first pool consists of neurons 1–10, second pool of neurons 11–20, etc.). Neuron pools were activated successively every 10 ms (first pool, followed by second pool 10 ms later, followed by third pool 10 ms later, etc.). Neurons within each pool fired synchronously within a time window *T*. For precise within-pool synchronization (left panels, *T* = 1 ms) strong unidirectional connections dominate in the resulting network (cf., Clopath et al., 2010, Figure 4), whereas for coarse synchronization (right panels, *T* = 5 ms) bidirectional connections dominate. Spike recordings show activity for neurons 1–30 during first 500 ms of simulations. All neurons fired with 10 spikes/s on average. Effective delays were *d* = 1 ms. Results for coarse synchronization with *T* = 10 ms were similar to results with *T* = 5 ms (not shown).

In the following we reproduce and extend one of the simulation experiments by Clopath and colleagues in order to show that the hypothesized one-to-one relation between connectivity and coding is rather unlikely to hold true (Figures 2 and 3). For this we have implemented the voltage-based STDP model and simulated a small network of eight neurons with complete recurrent synaptic connectivity including axonal transmission delays, as described by Clopath et al. (2010, Figure 4). Then we stimulated neurons in accordance with different assumptions on the neural code and evaluated the resulting modification of synaptic strengths.

**Figure 4:**
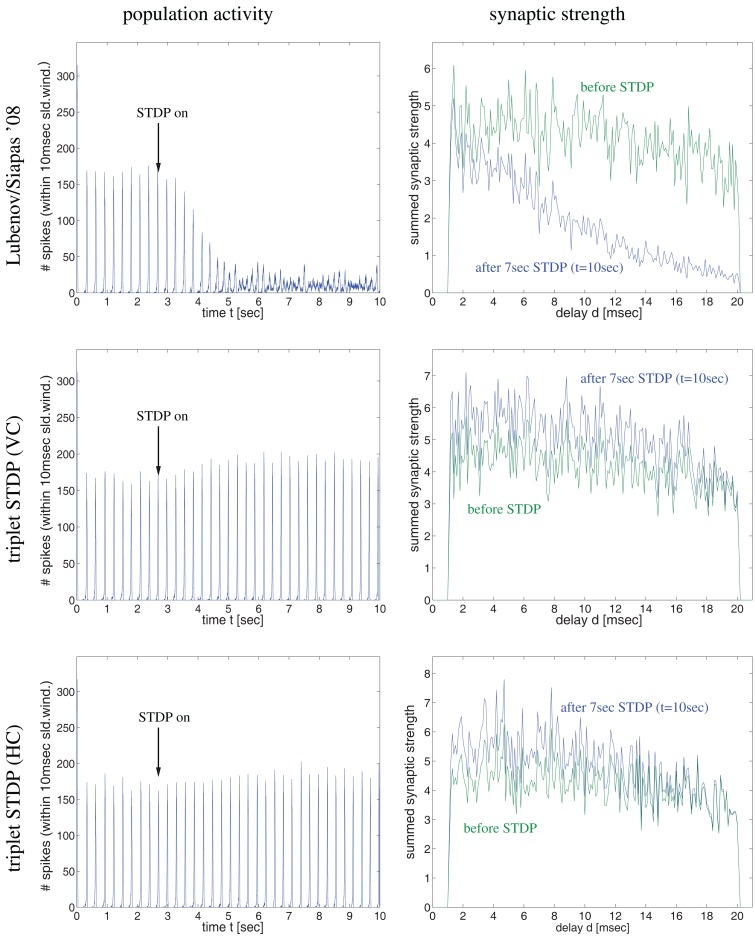
**Synaptic coupling and decoupling in a simple recurrent network model of 100 excitatory neurons with a broad distribution of axonal delays similar as described by Lubenov and Siapas (cf., Lubenov and Siapas, 2008, Figure 4)**. Left panels show spike activity within a 10 ms sliding time window summed over all neurons of a single simulation. Right panels show corresponding histograms for the distribution of summed synaptic strength over propagation delay *d* before switching on STDP at *t* = 3 s (green curve) and after 7 s simulation including STDP at *t* = 10 s (blue curve). Delays were drawn randomly from clipped Gaussians (mean ± s.d = 5 ± 20 ms, clipped to 1 ≤ *d* ≤ 20 ms). Top panels correspond to unbiased Hebbian doublet STDP (all-to-all linear model with *A*_+_ = *A_−_* = 0.5, τ_+_ = τ_−_ = 20 ms) similar as in (Lubenov and Siapas, 2008). Consistent with their results, oscillatory synchronization (*f* = 4 Hz) decouples neurons. Middle and bottom panels correspond to similar simulations but using more realistic triplet STDP models fitted to data from visual cortex (middle) and hippocampus (bottom). Here bidirectional couplings are even strengthened and oscillations are preserved. As in (Lubenov and Siapas, 2008), individual synaptic weights are clipped to the range 0 < *w* < 10 (initially *w* = 8 ± 0.8). Without clipping, synaptic weights (and oscillation amplitude) would increase even further for triplet STDP (data not shown).

Consistent with the results of Clopath and colleagues, our simulations confirm that strong bidirectional synaptic connections could be explained by a rate code (Figure 2A). Specifically, stimulation leads to LTD and LTP of synapses that connect neurons firing at low and high rates, respectively. In a second experiment, we repeatedly stimulated the neurons with strong but brief inputs such that all neurons had low average firing rates, but different subgroups of the neurons synchronized their spikes within time windows of different widths *T* (Figure 2B). Again consistent with the previous reports (Song and Abbott, 2001; Knoblauch and Sommer, 2003; Lubenov and Siapas, 2008; Clopath et al., 2010), it was possible to observe LTD and, thus, decoupling of neurons firing in synchrony. Surprisingly, decoupling occurred only if synchronization was very precise (*T* = 1 ms, cells 1–4), whereas synapses connecting coarsely synchronized neurons (intermediate *T* = 10 ms, cells 5–8) were potentiated and the corresponding neuron groups developed strong bidirectional connections. A similar result is actually visible, although not discussed, in Clopath et al. (2010, Figure 2 SA, lowermost matrix row).

Next we investigated synfire-chain-type sequential dynamics in a larger network of 100 neurons using the same voltage-based STDP model as before (Figure 3). For this, we divided neurons into ten pools of ten neurons and implemented a cyclic activation of neuron pools, similar as described by Clopath et al. (2010). Neurons within a pool were stimulated such that spikes were synchronized within a time window of width *T*. The time interval between stimulation of two successive pools was 10 ms such that one stimulation cycles lasts 100 ms. Again consistent with the results of Clopath and colleagues, precise spike synchronization of within-pool neurons (*T* = 1 ms) resulted in decoupling of synchronized within-pool neurons and a dominance of strong unidirectional between-pool connections (Figure 3, left panels; cf. Clopath et al., 2010; Figure 4). However, already slight increases of synchronization width toward more realistic values (e.g., *T* = 5 ms; see Discussion) resulted in a dominance of strong within-pool bidirectional connections over strong between-pool unidirectional connections (Figure 3, right panels).

Our results imply that strong bidirectional connections as observed in visual cortex are consistent with both rate coding and temporal coding based on coarse spike synchronization with sequential or non-sequential spatio-temporal correlations. This contradicts speculations that bidirectional connectivity would be evidence for rate coding and that synchronization would generally have a decoupling effect on coactivated neurons.

As the latter idea was emphasized in another influential study by Lubenov and Siapas (2008) we have also reevaluated their model in order to confirm our conclusion. In a first simulation experiment illustrated by Figure 4, we have implemented a network of 100 recurrently connected regularly spiking excitatory neurons with transmission delays (1 < *d* < 10 ms), similarly as described by Lubenov and Siapas (2008, Figures 4 and 7). Without STDP (0 < *t* < 3 s) neurons engage in slow collective oscillations around 4 Hz. As no inhibitory neurons are included, recurrent excitation induces high-frequency bursts where, during each oscillation period, each neuron emits multiple spikes until activity ceases due to habituation. At time *t* = 3 s, STDP is switched on. As previously reported by Lubenov and Siapas using an unbiased Hebbian STDP rule, we observed strong LTD of recurrent synapses leading to a decoupling of neurons and, correspondingly, a desynchronization of spike activity. However, for any biologically more realistic STDP rule, recurrent connections became even stronger (and oscillations were preserved), as shown for the triplet STDP model fitted to data from visual cortex (middle panels) or hippocampus (bottom panels). Similar results can be obtained even for the much simpler doublet STDP model if using plausible parameters that are compatible with experimental data (Figure S2 in Supplementary Material; see Discussion). Additional simulations confirmed that it is very difficult to obtain LTD for realistic STDP models and parameters under the described neural dynamics. We observed unequivocal decoupling and desynchronization only for modified neuronal habituation parameters preventing bursting and very precise spike synchronization that could be achieved only by applying precisely timed external low frequency stimulation (Figure S3 in Supplementary Material). This result is actually consistent with the therapeutic effects of deep brain stimulation in patients suffering from Parkinson’s disease (where we do not have to assume an abnormal STDP rule as suggested by Lubenov and Siapas, 2008, Figure 7).

In a further simulation we have investigated more realistic networks including excitatory and inhibitory conductance-based neuron models (for details see Knoblauch and Palm, 2001, 2002b) and triplet STDP models fitted to visual cortex data (Pfister and Gerstner, 2006; Morrison et al., 2008). Neurons are driven by external Poissonian stimulation such that initial firing rates are significantly above spontaneous level (approx. λ = 6 spikes/s). Initial synaptic weights are such that the network is in an asynchronous firing regime. When STDP is switched on (at time *t* = 3 s), synaptic strength generally increases independently of the delay (lower left panel, green curve for *t* = 5 s). With increasing synaptic strengths, activity becomes more and more synchronous and oscillatory (at about *f* = 10 Hz). Around *t* = 10 s (blue curves), synchrony is still coarse enough such that LTP dominates for almost any delay *d*. However, as synchronization becomes more and more precise, connections with large transmission delays become weaker, whereas connections with short delays still increase strengths (cyan curves). Even for very strong synaptic weights, synchronization is never precise enough to evoke LTD in short-delay connections. We observed corresponding results also for larger oscillation frequencies, STDP fitted to hippocampal data, and even simple doublet STDP models for plausible sets of parameters (see Figures S4, S5, and S2 in Supplementary Material).

To summarize our simulations, the decoupling force of STDP for synchronized neural activity seems not as general as emphasized in previous studies (Lubenov and Siapas, 2008; Clopath et al., 2010; cf. Figure 1). Instead, our simulations of plausible doublet, triplet, or voltage-based STDP models reveal that physiological spike synchronization consistently couples coactivated neurons by growing strong bidirectional connections even for large axonal propagation delays, synfire-type sequential neural dynamics, and low average firing rates. To understand and verify the generality of our results, the following sections present analyses and additional simulations for various doublet and triplet STDP models (Song and Abbott, 2001; Morrison et al., 2008) in order to determine conditions when synchronization of neural activity leads to either coupling or decoupling.

### 2.2 Theoretical conditions for coupling or decoupling of synchronized neurons

**Figure 5:**
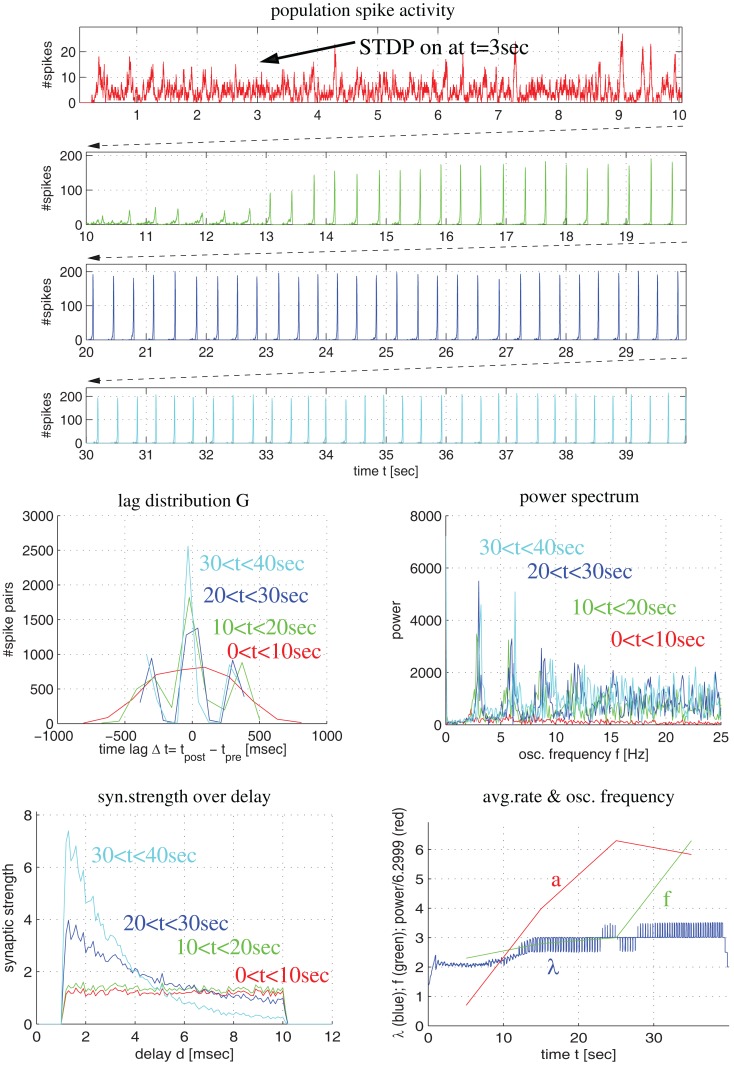
**Synaptic coupling in a more realistic recurrent network of excitatory and inhibitory conductance-based neurons using triplet STDP fitted to visual cortex**. Top panels show population spike recordings as function of time *t* (225 excitatory cells driven by external Poissonian stimulation). As in Figure 4, STDP is switched on at *t* = 3 s. Middle panels show lag distribution (middle left) and power spectrum (middle right) for different segments of the spike recordings (as indicated by colors). Bottom panels show distribution of synaptic strength as a function of delay (lower left) and average firing rate (λ), oscillation frequency (*f*) and oscillation amplitude (*a*) as a function of time (lower right). Note that, initially, the neurons fire asynchronously (red curves). STDP slowly increases recurrent synaptic strengths (green) until activity becomes oscillatory (*f* = 4 Hz) and coarsely synchronized. Then synaptic weights quickly increase, in particular, for connections with small delays (blue and cyan).

In recurrent networks, there is a mutual dependency between synaptic plasticity and neuronal dynamics. For example, in the simulations of Figure 5 uncorrelated spike activity above spontaneous level increased synaptic weights which caused an increase in spike synchrony and, in turn, an even stronger increase of synaptic strength. Previous studies have attempted to analyze this mutual dependency in a common theoretical framework (Levy et al., 2001), however, at the price of making numerous presumptions and approximations, for example, on network circuitry, neuronal firing patterns, plasticity model, and distribution of propagation delays. Moreover, generality of such approaches is further limited because neuronal dynamics will depend crucially on further assumptions on anatomy and plasticity of inhibitory circuits (Markram et al., 2004; Lu et al., 2007; Lamsa et al., 2010; Vogels et al., 2011)^1^. The following pursues a much simpler approach by separating the theory on weight change as a function of neuron dynamics from other theories on neuron dynamics as a function of synaptic strength. For the latter there are in fact already numerous prior results that analyze how firing dynamics depends on the strengths of excitatory and inhibitory synapses in simple recurrent networks (e.g., see Brunel, 2000). To complement these prior analyses, we compute mean weight change Δ*w* for different STDP models under the assumption of simple stationary firing regimes including synchronization, oscillations, and uncorrelated Poissonian firing. The benefit of this approach is that we can compute closed-form expressions for Δ*w* which, together with the previous theories, give us an overview of conditions when synchronization leads to either coupling or decoupling of coactivated neurons in simple circuits.

#### 2.2.1 Linear doublet STDP models

**Figure 6:**
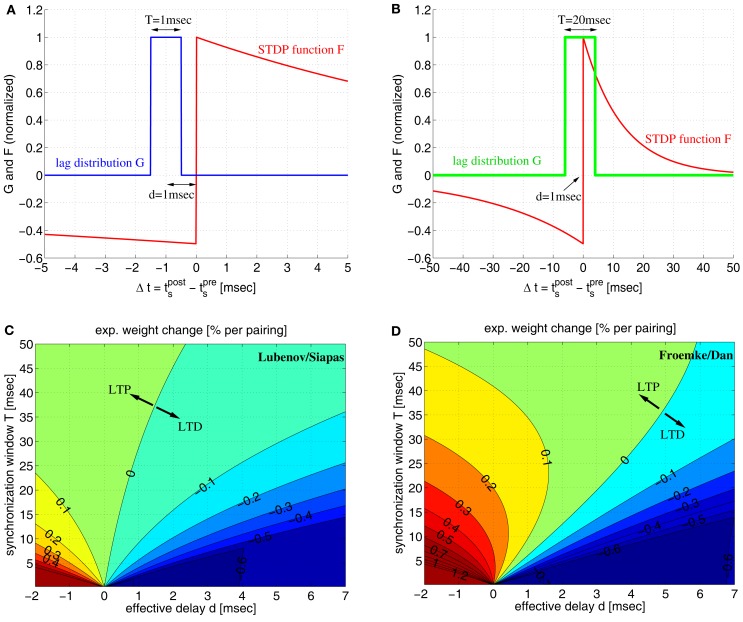
**Simple linear doublet STDP induces potentiation of synaptic weights for coarsely synchronized spike activity**. **(A)** Distribution *G*(Δ*t*) of time lags $\Delta t:=t_{s}^{\text{post}}-t_{s}^{\text{pre}}$ between presynaptic and postsynaptic spikes for a mean effective transmission delay *d* = 1 ms and precise spike synchronization where lags are uniformly distributed within a time window of width *T* = 1 ms. Plot shows also STDP function *F*(Δ*t*) corresponding to experimentally measured weight changes for spike pairings with lag Δ*t*. We assume *F*(Δ*t*) = A_+_exp(Δ*t*/τ_+_) for Δ*t* > 0 and *F*(Δ*t*) = −*A_*exp(Δ*t*/τ_−_) for Δ*t* < 0 with experimentally measured parameters *A*_+_ = 0.0147, *A*_−_ = 0.0073, τ_+_ = 13 ms, τ_−_ = 34 ms of (Froemke and Dan, 2002; see methods). Since *T* < 2*d* all spike pairs induce LTD. **(B)** Same as **(A)**, but for coarse synchronization with *T* = 10 ms. LTP is possible for *T* > 2*d* and *A*_+_ > *A*_−_ when the expected weight change per pairing, Δ*w*: = ∫*F*(*t*)*G*(*t*)*dt*, is positive. **(C)** Contour plot of expected weight change Δ*w*·100% [equation (10)] as a function of effective transmission delay *d* and synchronization window *T* using artificial STDP parameters similar as (Lubenov and Siapas, 2008; *A*_−_ = 0.0073, *A*_+_ = 1.1*A*_−_; τ_+_ = τ_−_ = 20 ms). LTD dominates for plausible *d* > 0 unless *T* is very large (corresponding to rate coding). LTD monotonically increases with decreasing *T* for any *d* > 0. Thus, for such parameters, synchronization decouples coactivated neurons. **(D)** Same as **(C)** but for realistic STDP parameters as in **(A)**. Still, there is strong LTD for precise synchronization (e.g., *T* < 5 ms). By contrast, for a wide parameter range, coarse synchronization (e.g., 5 ≤ *T* ≤ 50 ms) induces LTP even when assuming *d* ≈ *d*_ax_ and realistic axonal delays for local connections, 0.5 < *d*_ax_ < 5 ms (see also Figure S6 in Supplementary Material). For each *d* > 0 there is an optimal *T* that maximizes LTP (or minimizes LTD if *d* is very large). Thus, for realistic parameters, coarse synchronization consistently couples coactivated neurons.

In linear doublet STDP models (Gerstner et al., 1996; Song et al., 2000; Izhikevich and Desai, 2003; Morrison et al., 2008), the change of synaptic weight depends only on the time lags Δ*t*: = *t*_post_ − *t*_pre_ of relevant presynaptic and postsynaptic spike pairs according to an experimentally measured STDP function *F*(Δ*t*) (Figures 6A,B; cf., section 4.2). For cortical areas there are numerous reports of Hebbian STDP with LTP for Δ*t* > 0 (pre-before-post pairing), and LTD for Δ*t* < 0 (post-before-pre pairing; Bi and Poo, 1998; Froemke and Dan, 2002; Froemke et al., 2005). Typically, there is a sharp transition zone around zero time lag where LTP and LTD are maximal, *F*(ε) → A_+_ ≫ 0 and *F*(−ε) → −A_+_ ≪ 0 for 0 < ε → 0. For larger time lags, |Δ*t*| → ∞, it is typically assumed that LTP and LTD decay exponentially with time constants τ_+_ and τ_−_, respectively. Virtually all experiments demonstrate that the LTP amplitude is significantly larger than LTD amplitude, *A*_+_ ≫ *A*_−_, and that the time window for LTP is significantly shorter than for LTD, τ_+_ ≪ τ_−_ (Bi and Poo, 1998; Song et al., 2000; Froemke and Dan, 2002; Froemke et al., 2005). By contrast, many theoretical studies assume *A*_+_ = *A*_−_ and/or τ_+_ = τ_−_ to reduce the number of free parameters or to ease analyses (Levy et al., 2001; Morrison et al., 2007; Lubenov and Siapas, 2008). Unless noted otherwise, we use experimental parameters from Froemke and Dan, 2002; *A*_+_ = 0.0147, *A*_−_ = 0.0073, τ_+_ = 13 ms, τ_−_ = 34 ms).

If the distribution of the time lags, *G*(Δ*t*), is known, then the expected weight change is determined by the integral of the product of *F* and *G*. For all-to-all (AA-) STDP (Song and Abbott, 2001), all spike pairs contribute equally and *G* is essentially the cross correlation function of presynaptic and postsynaptic spike recordings (Singer and Gray, 1995). Thus, rate coding implies flat *G* for relevant time lags, whereas temporal coding by synchronization implies that *G* has a central peak. To simplify analysis we assume lag distributions with rectangular central peaks of width *T* shifted by effective transmission delays *d* = *d*_ax_ − *d*_bap_. We consider both positive and negative delays as axonal delays, *d*_ax_, and dendritic delays of the backpropagating action potential, *d*_bap_, may compensate each other, where small positive delays (e.g., *d* ≈ 1 ms) seem most realistic for local connections (Girard et al., 2001; Kampa and Stuart, 2006; see Figure S6C in Supplementary Material). A simple analysis gives closed-form expressions for the expected weight change, Δ*w* = ∫*F*(*t*)*G*(*t*)*dt*, as function of STDP parameters, transmission delay, and precision of synchronization [see equation (10)].

Figure 6 illustrates some results of the analysis for lag distributions with single peaks of width *T*. As expected from the previous simulations of realistic STDP, precise spike synchronization (small *T* → 0) induces LTD for realistic positive delays *d*. However, with decreasing precision of synchronization (i.e., increasing *T*) LTP becomes possible even for large *d* ≫ 0. The reason is that sufficiently large *T* > 2*d* allows the peak of the lag distribution *G* to overlap with the LTP window of the STDP function *F*. Then the expected weight change Δ*w* can become positive if the LTP amplitude *A*_+_ of *F* is significantly larger than the LTD amplitude *A*_−_, where the resulting LTP is strongest for small delays *d*. Still, particular parameter choices as by Lubenov and Siapas (2008, *A*_+_ ≈ *A_−_*, τ_+_ = τ_−_; see Figure 6C) can lead to the wrong ideas that LTP would be possible only in a very restricted parameter range, that LTP would monotonically decrease with smaller *T*, and that synchronization would have a generally destructive effect on connections between coactivated neurons. However, physiological experiments consistently demonstrate that *A*_+_ and τ_−_ are significantly larger than *A*_−_ and τ_+_, respectively (e.g., Bi and Poo, 1998) implying a much larger parameter range for LTP (Figure 6D). Specifically, realistic parameters imply a non-monotonic relation between weight change and precision of synchronization. For any reasonable positive delay *d*, there is an optimal synchronization window *T* that maximizes LTP (e.g., *T* ≈ 15 ms for *d* = 1 ms). For larger *T* or uncorrelated firing, LTP turns again to LTD because ∫*F*(*t*)*dt* < 0 for realistic parameters (e.g., Bi and Poo, 1998; Song and Abbott, 2001). For very large *d*, LTP is not possible, but there is still an optimal *T* minimizing LTD.

Thus, this simple analysis of doublet STDP confirms and explains our results obtained for realistic voltage-based STDP models. Further, it disproves the prejudice that simple doublet STDP models would be generally unable to produce bidirectional synaptic connections in recurrent networks (Gerstner et al., 1996; Song and Abbott, 2001; Kozloski and Cecchi, 2008). Finally, our findings reject the hypothesis that spike synchronization would generally exert a decoupling force on coactivated neurons for Hebbian STDP and realistic axonal transmission delays (Lubenov and Siapas, 2008). Instead, realistic STDP parameters imply an optimal time window (*T* ∼ 10 ms) where synchronization leads to maximal LTP and, thus, to bidirectional connectivity patterns in local recurrent networks, whereas decoupling occurs only for particular parameters not supported by experiments. The following subsections demonstrate the generality of our findings by investigating various further variants of doublet STDP.

#### 2.2.2 Weight dependence and non-linear doublet STDP models

For simple linear doublet models discussed so far, individual spike pairs add up linearly to total weight modification. This implies some obvious inconsistencies with experimental findings: For example, synaptic strength may diverge toward infinite values, *w* → ±∞, unless the range of synaptic strengths is artificially clipped to a fixed interval [0,*w*_max_] (e.g., Gerstner et al., 1996; Song et al., 2000; Izhikevich and Desai, 2003; Morrison et al., 2008). Moreover, there is evidence that weight modification depends on the absolute weight such that for increasing *w* the effect of LTP decreases whereas the effect of LTD increases (Liao et al., 1992; Bi and Poo, 1998; Montgomery et al., 2001; Wang et al., 2005; but see Sjöström et al., 2001 for negative results)^2^. Such weight dependence of STDP has led to non-linear models of STDP (e.g., van Rossum et al., 2000; Rubin et al., 2001; Gütig et al., 2003; Morrison et al., 2007, 2008).

We have analyzed two classes of non-linear doublet models, the power-law model of Morrison et al. (2007), and a model proposed by Gütig et al. (2003) that interpolates between additive and multiplicative models (Rubin et al., 2001). By extending the linear analysis to the non-linear model, we still can derive closed-form expressions for the equilibrium weight *w*_∞_ assuming rectangular lag distributions *G* and infinite simulation time [see equations (13 and 16)].

**Figure 7:**
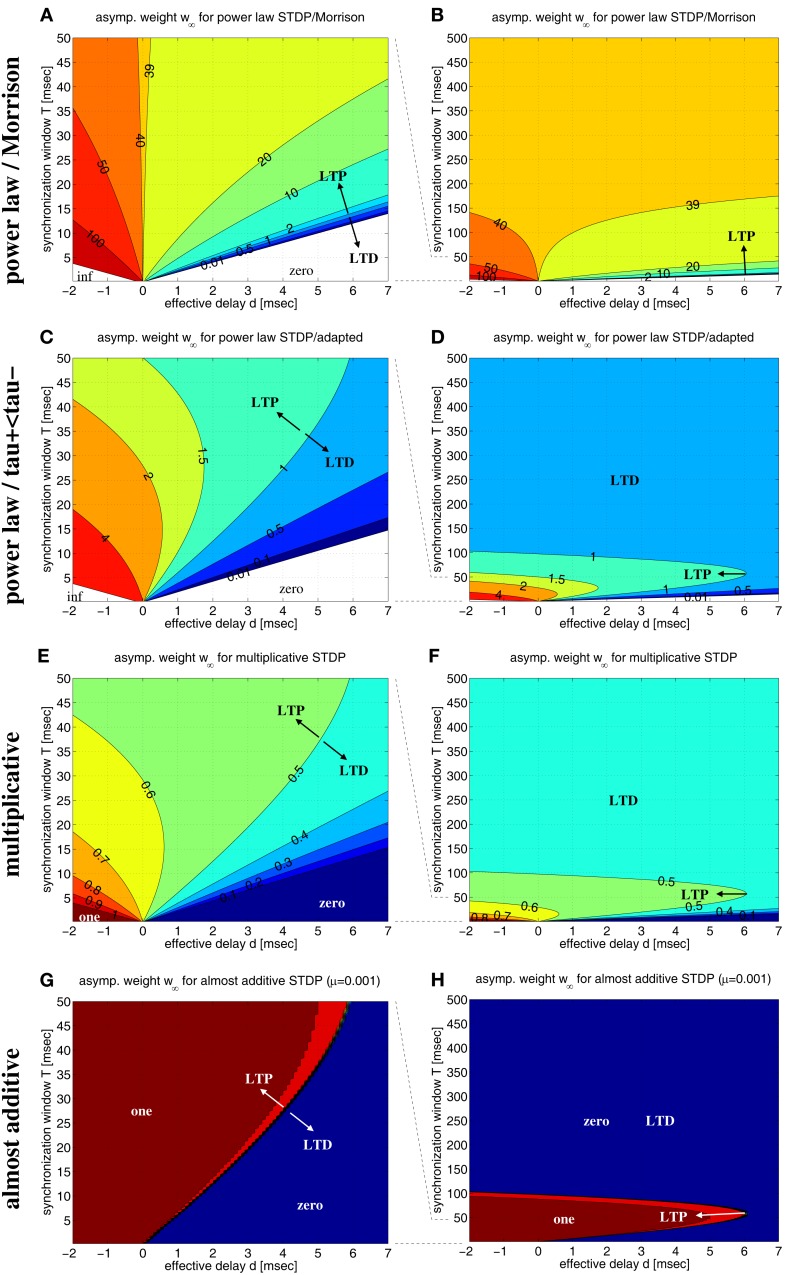
**Equilibrium synaptic weights *w*_∞_ for various non-linear doublet STDP models**. Contour plots show *w*_∞_ as function of effective delay *d* and synchronization window width *T* assuming rectangular lag distributions as in Figure 6. **(A)** Power-law STDP model [equation (11)] with original parameters from (Morrison et al., 2007; μ = 0.4, *w*_0_ = 1 pA, α = 0.11, τ_+_ = τ_−_ = 20 ms). Data computed from equation (13). **(B)** Same as **(A)**, but for a larger range of synchronization window width (0 < *T* < 500 ms). **(C,D)** Power-law model as in **(A,B)**, but with adapted parameters such that at the reference weight, *w* = *w*_0_, the resulting STDP function *F*(Δ*t*;*w*) is consistent with experimental data used for the additive model of Figure 6 (μ = 0.4, *w*_0_ = 1 pA, α = 0.0073/0.00147, τ_+_ = 13 ms, τ_−_ = 34 ms). **(E,F)** Multiplicative STDP [equation (15) with μ = 1] using parameters consistent with the additive model of Figure 6 (α = 0.0073/0.0147, τ_+_ = 13 ms, τ_−_ = 34 ms). Data computed from equation (16). **(G,H)** Almost additive STDP (equation (15) with μ = 0.001). Other parameters are as in panels **(E,F)**. LTP and LTD as indiciated by arrows refers to equilibrium synaptic weights being larger and smaller, respectively, than reference weights [*w*_0_ = 1 for panels **(A–D)**; *w*_0_ = 0.5 for **(E–H)**].

Figure 7 shows contour plots of *w*_∞_ as a function of synchronization window *T* and effective transmission delay *d*. We first investigated the model of Morrison et al. (2007) with their original parameters, in particular, *A*_+_ ≫ *A*_−_ and τ_+_ = τ_−_ (Figures 7A,B). This choice of parameters implies several peculiarities: First, due to *A*_+_ ≫ *A*_−_, there is a large parameter range of positive delays *d* where coarse synchronization (*T* ∼ 10 ms) implies large synaptic weights and, thus, stable coupling of coactivated neurons. Second, however, using equal time constants τ_+_ = τ_−_ implies again that there is a monotonic relation between synchronization window *T* and the resulting equilibrium weight *w*_∞_. For positive delays, *d* > 0, synaptic strength decreases with increasing precision of synchronization (smaller *T*) and finally vanishes for very precise synchronization. Thus, due to this monotonic relation, one may conclude that synchronization would have an inherently decoupling effect on coactivated neurons, similar as Lubenov and Siapas (2008) have done using the linear doublet model (cf., Figure 6C). Finally, for rate coding with large *T* → ∞ the equilibrium weight becomes independent of *T*, *d*, and the firing rate. The theoretical value *w*_∞_ ≈ 39.6 is consistent with network simulations of Morrison and colleagues that reveal unimodal small-variance distributions of synaptic weights (cf., Morrison et al., 2007, Figure 2; see also section 2.1).

Next, we have investigated the power-law model with more realistic parameters such that, at reference weight *w* = *w*_0_, weight modification matches the data of Froemke and Dan (2002), in particular, we used τ_+_ ≪ τ_−_ (cf., section 4.2.1). The resulting contour plots (Figures 7C,D) are qualitatively very similar to the corresponding linear model (cf., Figure 6D). In particular, for each positive delay *d*, there is an optimal synchronization window *T* that maximizes equilibrium weight *w*_∞_. Thus, these results confirm the coupling force of coarse synchronization (*T* on the order of 10 ms) for the power-law model.

Similar is true also for the non-linear doublet model of Gütig et al., 2003 (Figures 7E–H). By adjusting a parameter μ ∈ [0;1], this model can continuously interpolate between additive models (μ = 0) and multiplicative models (μ = 1). For realistic parameters (*A*_+_ ≫ *A*_−_; τ_+_ ≪ τ_−_), all models maximize equilibrium weights *w*_∞_ for coarse spike synchronization. While the multiplicative model, similar as the previous models, shows a rather gradual dependence of *w*_∞_ on synchronization window width *T* (see Figures 7E,F for μ = 1), coming close to the additive model implies sharp threshold-like transitions between minimal and maximal weights (see Figures 7G,H for μ = 0.001).

Thus, concerning the question whether Hebbian STDP couples or decouples synchronized neurons, our analyses show that non-linear doublet models behave qualitatively very similar as the linear doublet models. In particular, they confirm our hypothesis that, for realistic STDP parameters and transmission delays, spike synchronization exerts an inherently coupling force on coactivated neurons unless synchronization is unrealistically precise. So far, our conclusions are based on theoretical analyses that assume simple rectangular distributions of time lags. This simplifies analyses, but, similar as using Gaussian distributions (Lubenov and Siapas, 2008), is not a plausible model for lag distributions resulting from realistic spike trains (except for the limit *T* → ∞ and AA-STDP corresponding to rate coding with flat lag distributions). The next sections verify our qualitative analyses for more realistic spike trains.

#### 2.2.3 Dependence on rates and oscillation frequency: all-to-all versus nearest-neighbor doublet STDP models

Lag distributions with single central peaks as assumed in the previous sections are plausible only for low mean firing rates. Since synchronization in the brain occurs often together with states of increased neural activity, the following analyses are based on lag distributions computed from a two-state model of spike trains (see Figure 14; Section 4.1). This model assumes the existence of a background state where the neurons fire spontaneously at rate λ_0_ with Poissonian firing statistics (all following experiments assume λ_0_ = 1 spike/s). Additionally, there is an activated state where the neuron fires with a higher rate λ_1_. We further assume that activated states last for time *T* and are induced by stimulation events. For *non-oscillatory synchronization* stimulation events occur irregularly with event rate λ*_e_* (Poissonian statistics). For *oscillatory synchronization* we assume that stimulation events occur regularly with oscillation frequency *f* = λ*_e_*. In both cases, we can compute the resulting mean spike rate λ from λ_0_, λ_1_, *T*, and λ*_e_* or *f* (for details see Section 4.1). The following computes expected changes of synaptic strength, Δ*w*, dependent on λ, λ*_e_* (or *f*), *T*, and effective transmission delay *d* for different linear doublet STDP models.

**Figure 8:**
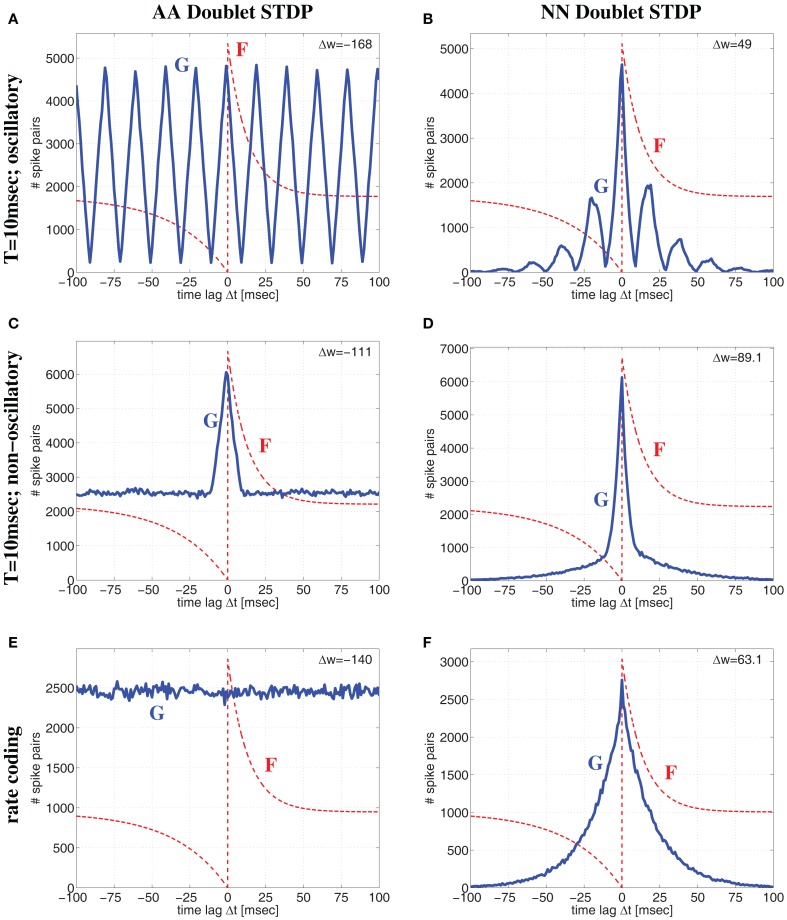
**Comparison of all-to-all (AA) and nearest-neighbor (NN) linear doublet STDP models for different regimes of neural activity for the Poissonian stimulation protocol**. Top panels **(A,B)** correspond to oscillatory synchronization with the Poissonian stimulation protocol of Figure 14 (see Section 4.1.1) for oscillation frequency *f* = 50 Hz, mean firing rate λ = 50 spikes/s, background firing rate λ_0_ = 1 spikes/s, synchronization window *T* = 10 ms, and effective delay *d* = 1 ms. Middle panels **(C,D)** show corresponding results for non-oscillatory synchronization (Section 4.1.2) but otherwise same parameters as for top panels (with an event rate λ_*e*_ = 50 events/s corresponding to *f*). Bottom panels **(E,F)** correspond to rate coding with uncorrelated Poissonian spike activity at λ = 50 spikes/s. Left panels **(A,C,E)** show lag distributions *G*(Δ*t*) (blue lines) of relevant spike pairings for AA-STDP and pre-/postsynaptic spike trains of length 100 sec. Note that *G* is essentially (up to time shift *d*) the correlogram of spike recordings (cf., Knoblauch and Hauser, 2011). Similarly, right panels **(B,D,F)** show lag distributions for NN-STDP. Lag histograms are slightly asymmetric in upper and middle panels because of non-zero delays *d* > 0 (cf., Figure 14). Each plot shows also the STDP function *F*(Δ) (red lines; same parameters as in Figure 6; curves are scaled and shifted for sake of visibility) and the resulting theoretical weight modification Δ*w* after 100 sec [upper right corner; equation (7)]. Note that NN-STDP predicts LTP (strongest for non-oscillatory synchronization), whereas AA-STDP yields LTD.

Besides the AA-model used in the previous sections here we will also consider nearest-neighbor (NN) variants of doublet STDP (van Rossum et al., 2000; Bi, 2002; Izhikevich and Desai, 2003; Burkitt et al., 2004) which is more consistent with frequency dependency of synaptic plasticity (Sjöström et al., 2001). For the all-to-all model (AA) of doublet STDP all spike pairs are equally relevant and contribute to the lag distribution *G*. In contrast, for nearest-neighbor (NN) STDP, for each presynaptic spike, only the both nearest postsynaptic spikes are relevant. Thus, for NN-STDP, the pairing distribution *G* is in general not equivalent to the cross correlation of the spike recordings. For example, for regularly oscillatory spike synchronization the lag distribution *G* will be oscillatory with multiple side peaks for AA-STDP (Figure 8A), whereas for NN-STDP the side peaks are masked by the nearest-neighbor constraint (Figure 8B). Similarly, for non-oscillatory synchronization, AA-STDP implies lag distributions composed of a single central peak and a significant flat contribution that extends far beyond the time window of STDP (Figure 8C), whereas lag distributions for NN-STDP are dominated only by the central peak (Figure 8D). Correspondingly, for rate coding with uncorrelated Poissonian firing, AA-STDP implies flat lag distributions (Figure 8E), whereas NN-STDP still shows single peaks (Figure 8F) that become narrow with high firing rate.

Thus, assuming realistic STDP parameters (*A*_+_ ≫ *A*_−_ and τ_+_ ≪ τ_−_) there is a fundamental difference between AA and NN-doublet models for larger average firing rates: The AA-model will always have lag distributions that maintain a significant fraction of large time lags favoring synaptic depression (since τ_+_ ≪ τ_−_) both for oscillatory and non-oscillatory firing^3^. By contrast, for any coding paradigm, the NN-model produces lag distributions that are dominated by narrow central peaks (still shifted by delay *d*) that favor synaptic potentiation (since *A*_+_ ≫ *A*_−_)^4^. Thus, even for realistic parameters, AA doublet STDP has a general tendency to underestimate LTP induced by synchronization, in particular for high firing rates or fast oscillations. This is visible in Figure 8 where AA-STDP yields strong LTD at λ = 50 spikes/s for Poissonian firing as well as for oscillatory and non-oscillatory synchronization (see also Figure S1 in Supplementary Material for analytical results). By contrast, NN-STDP yields strong LTP for all three regimes of neural dynamics. As found before for low average firing rate, LTP is strongest for (non-oscillatory) coarse synchronization.

**Figure 9:**
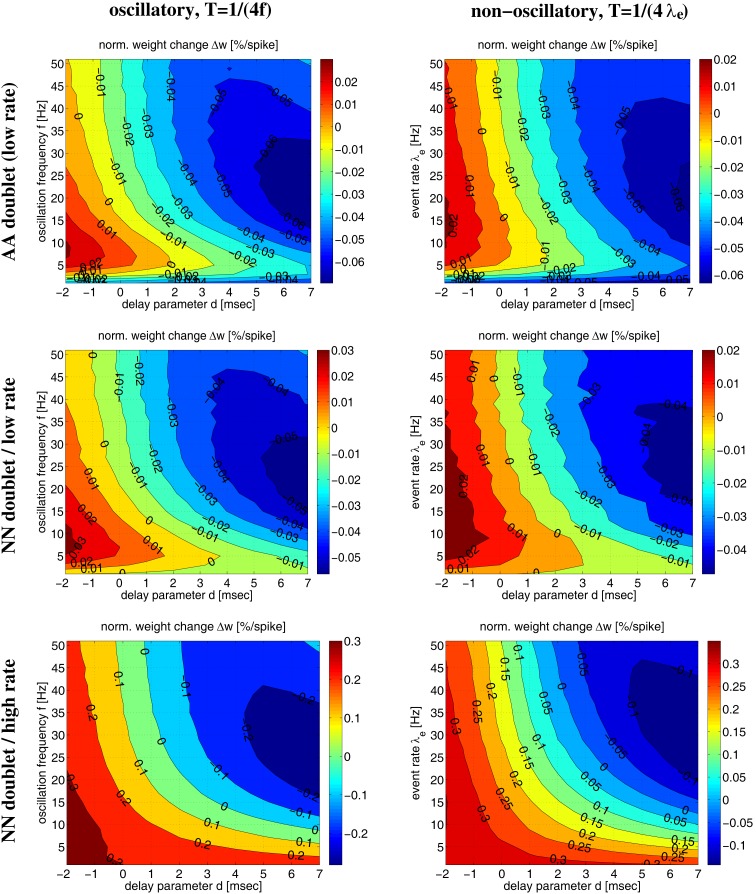
**Influence of oscillation frequency (or event rate) on weight modification for given average firing rate and linear AA/NN doublet STDP for Poissonian stimulation protocol (parameters as in Figures 6 and 8)**. Each contour plot shows weight modification Δ*w* (normalized) as a function of effective delay *d* and oscillation frequency *f* (or event rate λ_*e*_) for synchronization window *T* = 1/(4*f*) (or *T* = 1/(4λ_*e*_)). Left panels show results for synchronized oscillations with frequency *f* (see Figure 14, Section 4.1.1). Right panels show corresponding results for non-oscillatory synchronization with event rate λ_*e*_ (see Section 4.1.2). Top panels correspond to all-to-all (AA) doublet STDP. The plots show results for low mean firing rate λ = 4 spikes/s, but for AA-STDP results do not depend on λ up to scaling of Δ*w* (not shown). Middle panels correspond to nearest-neighbor (NN) doublet STDP at low firing rate λ = 4 spikes/s. Bottom panels correspond to NN doublet STDP at high firing rate λ = 30 spikes/s.

Figure 9 shows more systematic evaluations for oscillatory and non-oscillatory synchronization using the AA and NN-doublet models. Each plot shows expected weight change as function of transmission delay *d* and oscillation frequency *f* (or event rate λ*_e_*) for a given mean firing rate λ and assuming that spikes are synchronized within a quarter of the (mean) oscillation period, *T* = 1/(4*f*) or *T* = 1/(4λ*_e_*). For low average firing rate (λ = 4 spikes/s) AA and NN variants produce qualitatively similar results. For plausible delays *d* > 0, there is an optimal range of *f* (or λ*_e_*) around 5–10 Hz where LTP is maximal. For faster oscillations, LTP becomes weaker and turns to LTD. At high rates (λ = 30 spikes/s) the NN-doublet model increases the range and amplitude of LTP, and the optimal oscillation frequency comes close to zero. By contrast, for the AA-doublet-model the plots are independent of average firing rate up to scaling^5^. At least at higher firing rates, non-oscillatory synchronization yields more LTP than oscillatory synchronization^6^.

In summary, all doublet STDP models, if fitted to physiological data (*A*_+_ ≫ *A*_−_, τ_+_ ≪ τ_−_, relatively small effective transmission delays), can easily couple coarsely synchronized neurons (time scale *T* ∼ 10 ms). This disproves hypotheses put forward in recent studies that it would require realistic STDP models and rate coding to explain bidirectional connectivity patterns observed in cortical areas (Clopath et al., 2010), and that spike synchronization would generally exert a decoupling force on the synaptic connections between coactivated neurons (Lubenov and Siapas, 2008). The major reasons why earlier studies arrived at these wrong conclusions are as follows: First, to simplify analyses and to reduce the number of free parameters, many previous studies used simplified parameters sets, for example, *A*_+_ = *A*_−_ and/or τ_+_ = τ_−_ which are inconsistent with experimental findings (Song et al., 2000; Levy et al., 2001; Morrison et al., 2007; Lubenov and Siapas, 2008). Second, in many models spike synchronization is unrealistically precise which leads to strong LTD for synapses connecting coactivated neurons (Levy et al., 2001; Knoblauch and Sommer, 2003, 2004; Clopath et al., 2010). Third, many models used regimes of very regular oscillations which decreases LTP compared to more realistic irregular oscillations (Levy et al., 2001). Finally, many previous studies focused on the widely used linear AA-model variant which is inconsistent with the known frequency dependency of STDP (Gerstner et al., 1996; Levy et al., 2001; Song and Abbott, 2001; Knoblauch and Sommer, 2003, 2004; Kozloski and Cecchi, 2008; Lubenov and Siapas, 2008), and generally underestimates LTP unless spike activity is very low. By contrast, NN-doublet STDP models are much more consistent with the BCM-type (Bienenstock et al., 1982; Sjöström et al., 2001; Izhikevich and Desai, 2003) frequency dependency of STDP measured in physiological experiments. To further validate our results, the following section investigates a more realistic STDP model based on spike triplets that explicitly accounts for frequency dependency.

#### 2.2.4 Simulation and analysis of triplet STDP models

For simple doublet STDP models the weight change depends only on the time lags between relevant *pairs* of presynaptic and postsynaptic spikes. It has been argued that these models, in particular the AA variant, do not provide good fits to experimental data beyond simple low frequency pairing protocols. To verify our qualitative results obtained for doublet STDP, and to allow more quantitative predictions about the outcome of STDP for oscillatory and non-oscillatory synchronization with higher firing rates, the following considers the triplet model of Pfister and Gerstner (2006; see Section 4.4). For this model, synaptic weight change depends also on spike triplets in addition to doublets, and it has been shown to fit a much larger set of experimental data including non-linear dependencies on spike rates (Sjöström et al., 2001) as well as triplet and quadruplet experiments (Froemke and Dan, 2002; Wang et al., 2005).

Here our analyses and simulations focus on the nearest-neighbor (NN) variant of triplet STDP with parameters fitted to data from visual cortex or hippocampus (see Pfister and Gerstner, 2006, Tables 3 and 4). Similar results can also be obtained for the all-to-all (AA) variant of triplet STDP (data not shown). In fact, Pfister and Gerstner have shown that, unlike doublet STDP, the NN and AA variants of triplet STDP models are basically equivalent in explaining the available experimental data (see Pfister and Gerstner, 2006, Tables 3 and 4). For comparison, the following shows also results from simulating the NN-doublet model fitted to data from visual cortex (Froemke and Dan, 2002). Unless stated otherwise, all simulation experiments employ the Poissonian stimulation protocol described in the previous section (cf., Figure 14; see Section 4.1).

**Figure 10:**
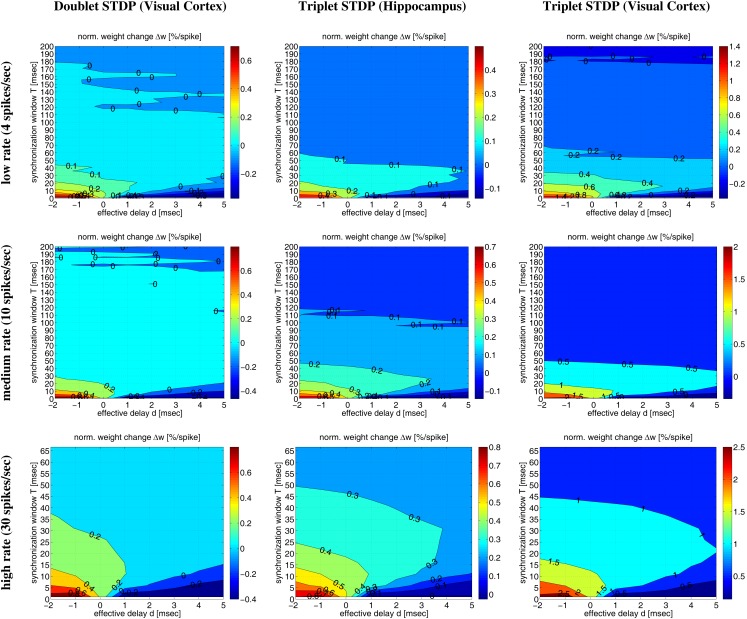
**Contour plots of expected weight change Δ*w* for NN-doublet and -triplet models**. Plots show Δ*w* (per postsynaptic spike; initial weight *w* = 1) as a function of synchronization window width *T* and effective transmission delay *d* corresponding to low activity (top panels; λ = 4 spikes/s), medium activity (middle panels; λ = 10 spikes/s), and high activity (bottom panels; λ = 30 spikes/s). Simulations implemented linear NN doublet STDP fitted to data from visual cortex (left panels; see Section 4.2; parameters from Froemke and Dan, 2002; see Materials and Methods), NN-triplet STDP fitted to data from hippocampus (middle panels; see Section 4.4.1), NN-triplet STDP fitted to data from visual cortex (right panels; see Section 4.4.1). All simulations used the Poissonian stimulation protocol described in Section 4.1.2 (non-oscillatory synchronization; λ/λ_*e*_ = 2; λ_0_ = 1 Hz).

Figure 10 shows contour plots of the expected weight change Δ*w* (per postsynaptic spike) as a function of the synchronization window width *T* and the effective delay *d* (similar to Figures 6 and 7) for low, medium, and high firing rates λ. The simulations confirm the main conclusions drawn from the simulations so far. First, for each transmission delay *d* there is an optimal synchronization window width *T* for which synaptic potentiation is maximized. Second, for realistic positive *d* the optimal *T* is in an intermediate range corresponding to coarse synchronization (on the order of *T* ∼ 10 ms). Third, precise synchronization (*T* < 5 ms) or uncorrelated firing (as expected for rate coding; *T* ≫ 50 ms) typically produces LTD unless firing rates are very high. There are some minor differences between the models and stimulation protocols (data not shown). For example, non-oscillatory synchronization yields more LTP than oscillatory synchronization, and LTP increases with the λ/λ*_e_* (or λ/*f*) ratio (see below). Otherwise, the NN-doublet model produces qualitatively very similar results as the two triplet model variants.

**Figure 11:**
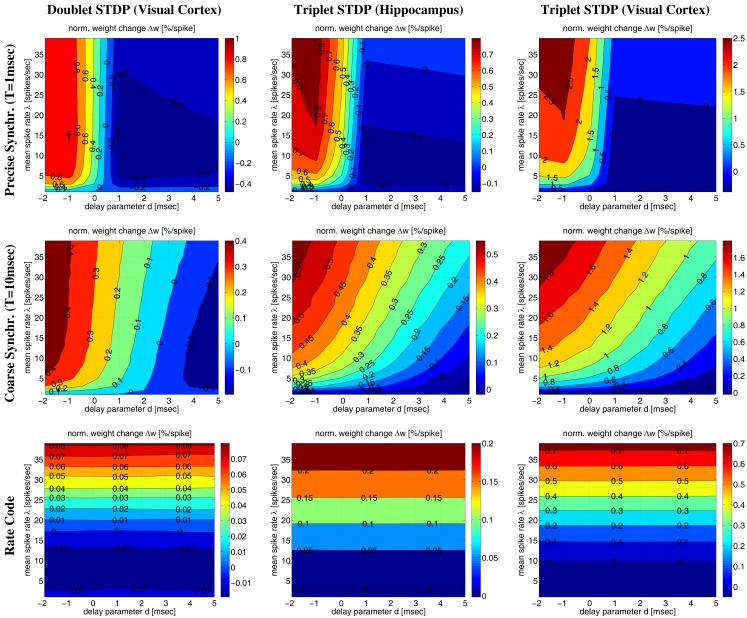
**Contour plots of expected weight change Δ*w* for NN-doublet and -triplet models**. Plots show synaptic weight change Δ*w* (per postsynaptic spike; initial weight *w* = 1) as a function of mean firing rate λ and effective transmission delay *d*. Panels correspond to precise spike synchronization (top panels; synchronization window *T* = 1 ms), coarse spike synchronization (middle panels; *T* = 10 ms), and rate coding (bottom panels; Poissonian firing). Simulations implemented NN doublet STDP fitted to data from visual cortex (left panels; see Section 4.2), NN-triplet STDP fitted to data from hippocampus (middle panels; see Section 4.4.1) NN-triplet STDP fitted to data from visual cortex (right panels; see Section 4.4.1). All simulations used the Poissonian stimulation protocol described in Section 4.1 (non-oscillatory synchronization; λ/λ_*e*_ = 2; λ_0_ = 1 Hz).

**Figure 12:**
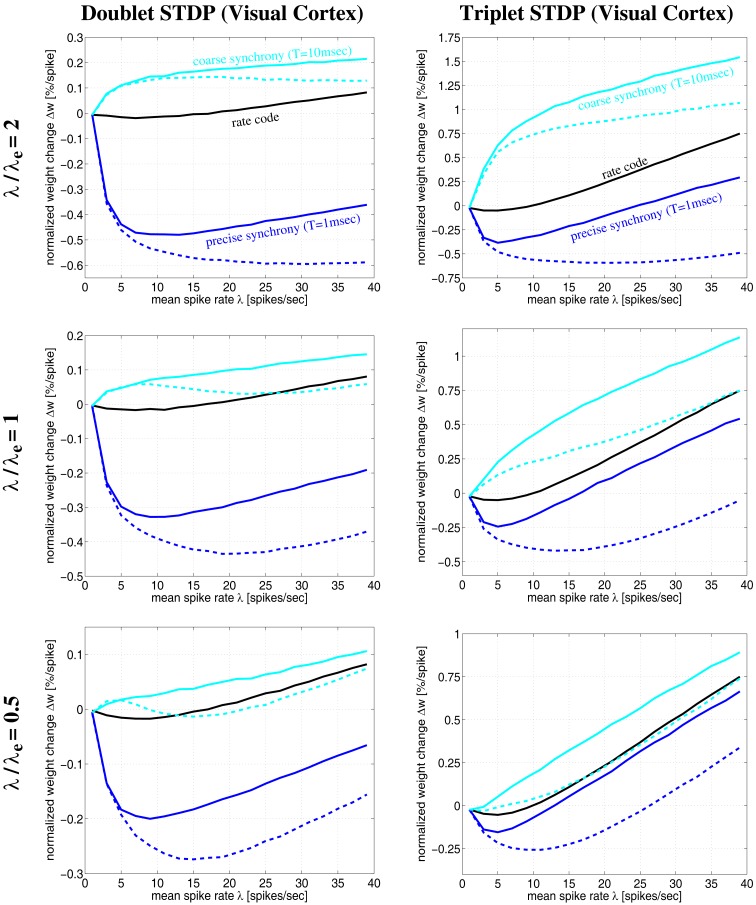
**Plots of expected weight modification Δ*w* for NN-doublet and -triplet models as function of mean firing rate λ using the Poissonian stimulation protocol**. Plots show results for different neural coding regimes and different STDP models fitted to data from visual cortex. Left panels correspond to nearest-neighbor doublet STDP (same parameters as in Figures 8–11). Right panels correspond to triplet STDP (same parameters as in Figures 10 and 11). Different curves correspond to precise synchronization within a time window of *T* = 1 ms (blue), coarse synchronization with *T* = 10 ms (cyan), and rate coding with uncorrelated Poissonian firing (black). Results are shown for regular oscillatory (dashed; frequency *f*) and non-oscillatory synchronization (solid lines; event rate λ_*e*_) assuming effective delay *d* = 1 ms and background firing at λ_0_ = 1 Hz (see Section 4.1). Note that coarse synchronization boosts synaptic potentiation at low firing rates. Doublet and triplet models yield qualitatively similar results.

Corresponding results are also visible in Figure 11 illustrating Δ*w* as function of average firing rate λ and propagation delays *d* for various firing regimes (whereas Figure 12 assumes a fixed delay *d* = 1 ms). For precise synchronization (small *T* = 1 ms, top panels) synaptic weights get generally depressed for realistic positive delays unless firing rates are very high. Although we assumed λ/λ*_e_* = 2 minor LTP occurs only for λ > 20 Hz (visual cortex) or λ > 30 Hz (hippocampus)^7^. For coarse synchronization (intermediate *T* = 10 ms, middle panels), by contrast, it is possible to obtain significant potentiation of synaptic weights even for realistic transmission delays and low firing rates. In fact, coarse synchronization at low rates near spontaneous activity is similarly effective for synaptic potentiation as employing uncorrelated Poissonian spike firing at high rates (as expected for rate coding; bottom panels).

The simulations of Figures 10 and 11 assumed a particular firing regime induced by non-oscillatory stimulation with an average spike count per stimulation event of λ/λ*_e_* = 2 (see Section 4.1.2). Qualitatively similar figures can be obtained for other firing regimes (data not shown; cf., Knoblauch and Hauser, 2011). Figure 12 shows some results for oscillatory and non-oscillatory firing and different λ/λ*_e_* for delay *d* = 1 ms. It can be seen that coarsely synchronized (*T* = 10 ms; cyan lines) non-oscillatory (solid lines) firing with bursting (λ/λ*_e_* = 2, top panels) is most effective for inducing strong LTP and coupling of coactivated neurons. By contrast, to induce strong LTD and decoupling, it is most effective to have precisely synchronized (*T* = 1 ms; blue lines) oscillatory (dashed lines) firing with bursting (top panels). At low firing rates, oscillatory and non-oscillatory firing is nearly equivalent. However, for coarse synchronization at high firing rates, non-oscillatory firing strongly increases LTP compared to oscillatory firing. Similarly, for precise synchronization at high rates, non-oscillatory firing strongly decreases LTD compared to regular oscillatory firing.

It should be noted that, due to neuronal refractory periods, the assumption of bursting (e.g., λ/λ*_e_* = 2) within the time window of precise synchronization (e.g., *T* = 1 ms) may be implausible for most neuron types. Nevertheless, smaller λ/λ*_e_* ≤ 1 (middle and bottom panels) yield similarly strong decoupling for precisely synchronized oscillations. Although LTP is somewhat reduced compared to bursting, coarse synchronization has still a strong coupling force on coactivated neurons even at low rates. Rate coding with Poissonian firing statistics (black lines) can also induce strong LTP, however, only at the price of much higher firing rates and, thus, much higher energy expenditures (Attwell and Laughlin, 2001; Laughlin and Sejnowski, 2003; Lennie, 2003)^8^.

**Figure 13:**
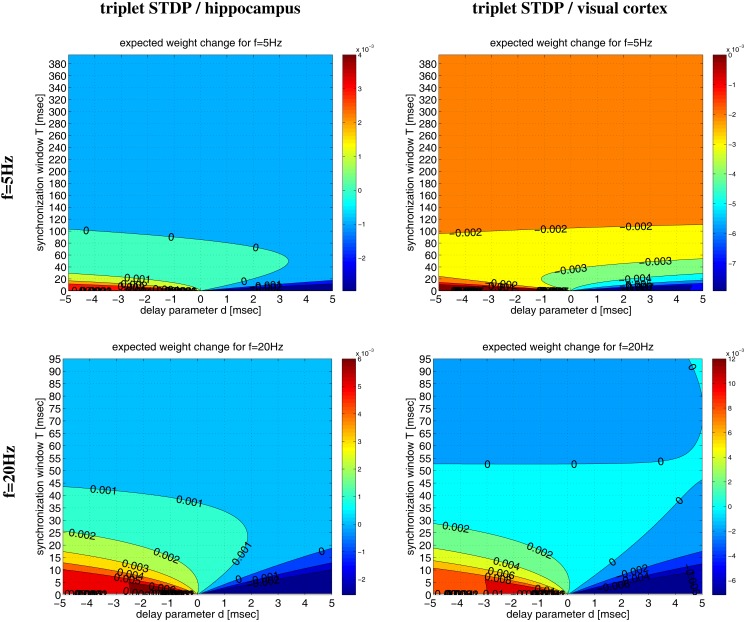
**Analytical weight change Δ*w* for regular oscillations**. Contour plots show expected weight change Δ*w* per pairing as computed from equation (22) as a function of effective delay *d* and synchronization window *T* for NN-triplet STDP with different oscillation frequencies *f* (being identical to firing rates λ). Data is shown for *f* = λ = 5 Hz (top panels) and *f* = λ = 20 Hz (bottom panels). Model parameters are fitted to data from hippocampus (left panels) or visual cortex (right panels) as described by Pfister and Gerstner (2006, Table 4, minimal models), see Section 4.4 for details.

**Figure 14:**
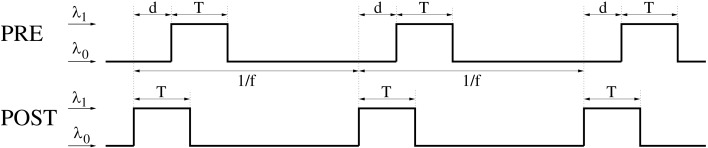
**General Poissonian stimulation protocol to evaluate modification of synaptic weights for oscillatory synchronization**. Neurons are synchronously stimulated for a time period of length *T* where they fire with rate λ_1_. Stimulation periods are repeated with frequency *f* (corresponding to an oscillation period 1/*f*). Without stimulation, neurons fire at a background rate λ_0_. Signal transmission causes an effective transmission delay *d* at the synaptic site. At time *t* = *idt* a spike is emitted with probability λ(*t*)*dt* where λ(*t*) is the firing rate at time *t*, *dt* = 0.1 ms is the simulation step size, and *i* = 0,1,2…. Similar stimulation protocols are used for non-oscillatory synchronization and uncorrelated firing (see Section 4.1 for details).

**Figure 15:**
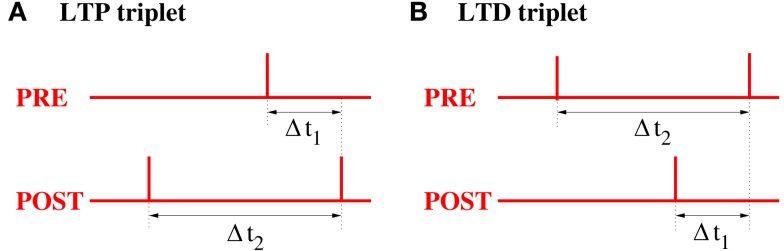
**Relevant time lags Δ*t*_1_ and Δ*t*_2_ between presynaptic and postsynaptic spikes (measured at the synaptic location) for NN-Triplet STDP**. **(A)** Potentiation (LTP) occurs after each postsynaptic spike as a function of the time lag Δ*t*_1_ to the previous presynaptic spike and the time lag Δ*t*_2_ to the previous postsynaptic spike [see equation (18)]. **(B)** Depression (LTD) occurs after each presynaptic spike as a function of the time lag Δ*t*_1_ to the previous postsynaptic spike and the time lag Δ*t*_2_ to the previous presynaptic spike [see equation (19)].

**Figure 16:**
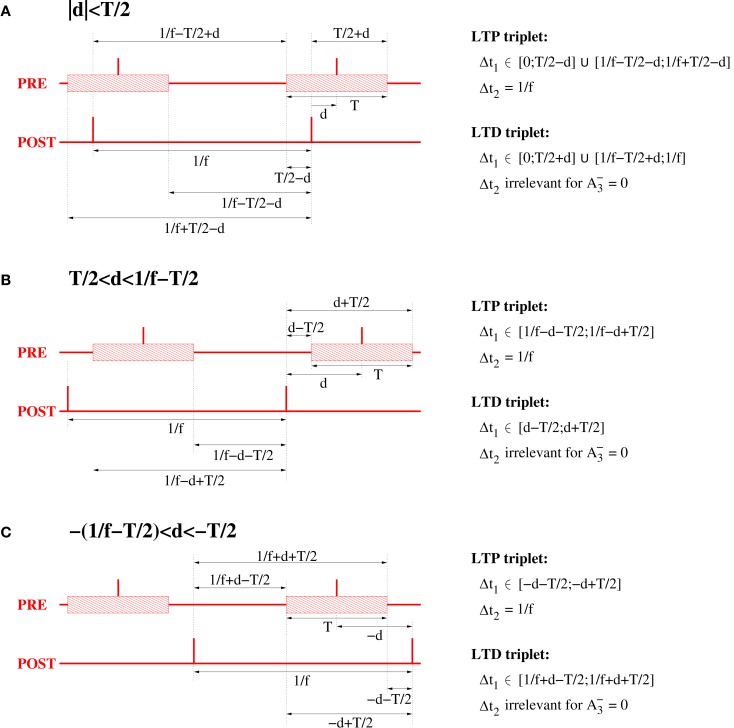
**Relevant triplet configurations for computing expected weight change for NN triplet STDP and oscillatory synchronization**. The plots assume oscillation frequency *f* and effective transmission delays *d*. For analytical ease, it is assumed that the postsynaptic neuron has constant inter-spike-intervals of length 1/*f*. The spikes of the presynaptic neuron are coarsely synchronized with the spikes postsynaptic neuron within a time window of width *T* (interval indicated by shaded blocks). **(A)** Spike configuration for small absolute delays, |*d*| < *T*/2. **(B)** Spike configuration for large delays, *T*/2 < |*d*| < 1/*f* − *T*/2. **(C)** Spike configuration for extremely negative delays, −1/*f* + *T*/2 < *d* < −*T*/*2*.

Even for λ/λ*_e_* ≤ 1 and coarse synchronization, it may seldom occur for our Poissonian stimulation model (see Section 4.1) that a neuron fires two or more spikes within its small absolute refractory period. However, because they are seldom, these bursts will have only a minor influence on the resulting weight change. We have verified this arguments by an analysis of the NN-triplet model assuming oscillatory firing where each neuron emits exactly one spike per oscillation period (corresponding to λ/λ*_e_* = 1; see Figure 16). This analysis provides a closed-form expression for the expected weight change given triplet STDP parameters, delay, precision of synchronization, and oscillation frequency [see equation (22)]^9^. The analytical results are well consistent with the Poissonian simulation experiments described above. Figure 13 shows data obtained from evaluating equation (22) to compute expected weight change Δ*w* as function of synchronization window *T*, effective propagation delay *d*, and oscillation frequency *f* (which is here identical to firing rate λ). These theoretical results are qualitatively similar to the results obtained from the more general “Poissonian” stimulation protocols (see Figures 10–12; cf., Figure 14), for example, coarse synchronization generally increases Δ*w* compared to precise synchronization or uncorrelated firing. However, there is significantly less LTP compared to the previous stimulation protocols. In particular, for visual cortex parameters and low activity (*f* = 5 Hz, upper right panel) it is impossible to get LTP for positive propagation delays. This discrepancy can be attributed to several factors. Most importantly, due to *A*_2+_ = 0, LTP can occur only for spike triplets (parameter *A*_3+_ > 0) if postsynaptic interspike intervals Δ*t*_2_ are sufficiently small (Figure 15, left). For Poissonian stimulation small Δ*t*_2_ occur due to occasional bursting (with multiple spikes per cycle even for λ ≤ *f*), whereas the “exactly one-spike-per-cycle” protocol prevents bursts and, thus, LTP^10^. Other factors include the absence of spontaneous activity outside the stimulation events of length *T* and the very precise “jitterless” oscillation of the postsynaptic cell^11^.

## 3 Discussion

We have simulated and analyzed various STDP models in order to derive conditions when STDP leads to either coupling or decoupling of neurons firing in synchrony. Our results demonstrate that STDP consistently couples synchronized neurons if key model parameters fit physiological data.

1. Synaptic potentiation must be significantly stronger than synaptic depression for small (positive or negative) time lags between presynaptic and postsynaptic spikes, e.g., *A*_+_ significantly larger than *A*_−_ for doublet models.
2. Spike synchronization must be sufficiently imprecise, for example, within a time window of *T* = 5–50 ms instead of *T* = 1 ms.
3. Axonal propagation delays should not be much larger than dendritic delays.

Condition 1 guarantees that averaging weight change Δ*w* over time lags Δ*t* around zero will result in significant LTP. It is supported by much of the available data using low frequency stimulation protocols (Bi and Poo, 1998; Bi and Wang, 2002; Froemke and Dan, 2002; Froemke et al., 2005; Dan and Poo, 2006). Some experiments, however, report a pronounced BCM-type frequency dependency of LTP and LTD amplitudes where there is only LTP (*A*_−_ < 0) at high stimulation rates, and only LTD (*A*_+_ < 0) at low rates (Bienenstock et al., 1982; Sjöström et al., 2001; Izhikevich and Desai, 2003). The latter seems to generally exclude coupling of synchronized neurons firing at low rates and is also reflected by more realistic triplet STDP models, for example, when stimulating with one spike per stimulation event at a low event rate (Figure 13)^12^. Nevertheless, more realistic firing patterns that include occasional bursting during brief and coarsely synchronized firing events (with high instantaneous firing rates) are sufficient to restore strong LTP even at low average firing rates near spontaneous activity (Figures 11 and 12)^13^. Strong LTP is also possible for uncorrelated Poissonian firing, however, only at the price of much higher firing rates and, thus, much higher energy expenditures (Attwell and Laughlin, 2001; Laughlin and Sejnowski, 2003; Lennie, 2003).

Condition 2 of coarse synchronization is necessary to escape strong LTD that would occur for very precise synchronization (*T* → 0) due to positive propagation delays *d* > 0 as illustrated by Figures 1 and 6. In fact, the assumption of coarse synchronization is uncontroversial since experiments report synchronization at zero time lag only when averaging over several synchronization events, whereas individual synchronization events can have significant time lags well on the order of 10 ms (Singer and Gray, 1995; Eckhorn et al., 2001; Yen et al., 2007). Similarly, in simulations of plausible recurrent network models it is impossible to obtain arbitrarily precise synchronization (even for extremely strong recurrent synaptic weights) due to propagation delays, finite synaptic strengths, and neuronal integration properties (see Figure 5; Knoblauch and Palm, 2002a). Only for strong and precisely timed feed-forward or external stimulation at low rates it appears possible to achieve the synchronization precision necessary for induction of strong LTD (Figure S3 in Supplementary Material; Lubenov and Siapas, 2008; Benabid et al., 2009).

Condition 3 of small effective propagation delays *d* is also necessary to escape strong LTD. For local connections (e.g., within a macrocolumn of 1 mm diameter) axonal delays *d*_ax_ of the majority of connections lie in the range of 0.5–5 ms (Girard et al., 2001). Because there are similar dendritic delays for the backpropagating action potential in the postsynaptic cell (Kampa and Stuart, 2006), the effective delays relevant for STDP may be quite small, e.g., 0–2 ms for the majority of local connections (see Figures S6 and S7A in Supplementary Material). By contrast, for more distant neurons or neurons from different cortical layers, *d*_ax_ and *d*_bap_ should strongly differ. For neurons from different cortical layers, the asymmetry in *d*_ax_ and *d*_bap_ would support unidirectional rather than bidirectional connections for synchronous firing at low rates (Figure S7B in Supplementary Material). Even then, strong bidirectional coupling would still be possible for larger average firing rates. For horizontally more distant cells, increasing effective delays could be compensated for by coarser synchronization with increasing *T* as described in experiments and network simulations (Eckhorn et al., 2001; Knoblauch and Palm, 2002a)^14^.

There is actually another implicit assumption (made by virtually all STDP models) that all spike pairings would contribute linearly to weight change on the *short* time scale of plasticity induction, whereas there is evidence that the initial phase of LTP or LTD is highly non-linear involving transitions between a small number of discrete synaptic states (Petersen et al., 1998; Montgomery and Madison, 2004; O’Connor et al., 2005). As current experimental protocols for investigating STDP test each synapse by repeated spike pairing with only one fixed time lag one may question the relevance of these experiments for predicting weight change for coarsely synchronized pairings. Therefore, future experiments should directly test individual synapses with distributed time lags, for example, randomly drawn from an interval [−*T*/2; *T*/2] (see also Supplementary Material).

Our results bear several important implications: First, they disprove an established prejudice that temporal coding by spike synchronization would be generally incompatible with STDP and bidirectional synaptic connectivity (Gerstner et al., 1996; Song and Abbott, 2001; Knoblauch and Sommer, 2003; Kozloski and Cecchi, 2008; Lubenov and Siapas, 2008; Clopath et al., 2010). This prejudice originates from the intuition that, due to the asymmetric temporal profile of STDP, spike synchronization would necessarily decouple coactivated neurons (as illustrated by Figure 1) and received some confirmation from earlier simulation studies using the AA-doublet STDP model to demonstrate the instability of bidirectional synaptic connections (Knoblauch and Sommer, 2003; Clopath et al., 2010), the decoupling force of spike synchronization (Lubenov and Siapas, 2008), and the self-organization of neuron dynamics into synfire-type sequential neuron dynamics with a domination of strong stable unidirectional connections (Levy et al., 2001). In the light of our study, these previous results appear as artifacts from using the simple AA-doublet STDP model with unrealistic parameters (e.g., *A*_+_ = *A*_−_, τ_+_ = τ_−_) and/or unrealistically precise synchronization (e.g., *T* ≪ 5 ms) and seem unlikely to hold when using more realistic STDP models (see Section 2.1). By contrast, we have shown for various simple and realistic model variants that STDP will typically stabilize bidirectional connections between synchronized neurons if the above mentioned conditions are fulfilled.

Second, our results show that network connectivity will not be as closely related to the underlying neural code as discussed in prior works (Clopath et al., 2010). In particular, we have shown that a dominance of bidirectional synaptic connections is not a reliable indicator of rate coding as such connectivity patterns can result as well from synchronous or sequential temporal codes (Figures 2 and 3) Neither is dominance of unidirectional connections a reliable indicator of a sequential temporal code, because unidirectional connections can result as well from (non-sequential) synchronization, for example, if axonal and dendritic delays are asymmetric as may occur for synapses connecting neurons from different cortical layers (see Figure S7B in Supplementary Material)^15^.

Third, we give an answer to the open question formulated by Fell and Axmacher (2011) how unequivocal LTP is accomplished by zero-lag phase synchronization. As explained above, the key insight is that coarse spike synchronization has a strong unequivocal coupling force on coactivated neurons for any plausible model parameters. Mutual coupling will be strongest if the time window of synchronization is in an intermediate range of perhaps 5–50 ms, whereas LTP is not possible for very precise synchronization or uncorrelated firing at low rates. Such coarsely synchronized spike activity is indeed consistent with irregular firing observed in numerous studies of cortical dynamics and natural stimulation which has been attributed to a “high conductance” activity state where excitation and inhibition are approximately balanced (e.g., Shadlen and Newsome, 1994; Vogels et al., 2005). Coarse spike synchronization fits as well to response properties of cortical neurons under natural stimulation (DeWeese and Zador, 2006; Yen et al., 2007; Jadhav et al., 2009) and the required synchronization window is consistent with a number of well known physiological time windows including duration of postsynaptic potentials, neuronal integration time constant, gamma oscillation period, and optimal peer prediction time (cf., Buzsaki, 2006, p. 163).

Thus, our results reconcile STDP with experimental findings that fast synchronized oscillations reflect feature integration (Singer and Gray, 1995), attention (Fries et al., 2001), and associative learning (Miltner et al., 1999), which suggests a constructive rather than destructive role of spike synchronization for memory (Jutras and Buffalo, 2010; Fell and Axmacher, 2011) and the formation of Hebbian cell assemblies (Hebb, 1949; Braitenberg, 1978; Palm, 1982; Knoblauch and Palm, 2002a; Lansner, 2009; Buzsaki, 2010). Moreover, a better understanding of conditions when spike synchronization leads to either coupling or decoupling of coactivated neurons may help to develop improved (e.g., deep brain) stimulation protocols for the therapy of diseases such as epilepsy, tinnitus, and Parkinson (Lubenov and Siapas, 2008; Benabid et al., 2009; Pfister and Tass, 2010).

## 4 Materials and Methods

### 4.1 Poissonian stimulation protocols for simulation experiments

To investigate the effect of STDP as a function of synchronization window *T*, effective propagation delay *d*, and mean firing rate λ we used the following neuronal stimulation protocol (cf., Figure 14).

1. Neurons generally have Poissonian firing characteristics.
2. Without any stimulation, neurons fire at a background rate λ_0_ = 1 spike/s.
3. During a stimulation event of length *T*, neurons fire with an increased rate λ_1_ > λ_0_.
4. Stimulation events are synchronized for presynaptic and postsynaptic neurons. That is, presynaptic and postsynaptic neurons increase firing rates during the same time interval of length *T*.
5. Effective propagation delay at the synaptic site is *d*. That is, if presynaptic and postsynaptic spikes occur synchronously in the cell soma, then the presynaptic spike lags by time *d* behind the postsynaptic spike at the location of the synapse.

#### 4.1.1 Oscillatory synchronization

To investigate firing regimes of oscillatory synchronization we additionally assume that stimulation events occur repetitively with oscillation frequency *f*. That is, the time difference between onsets of two successive stimulation events is always 1/*f* as illustrated by Figure 14. Then the neurons have a mean firing frequency

(1) $$\lambda =\frac{T}{1∕f}\lambda_{1}+\frac{1∕f-T}{1∕f}\lambda_{0}\;.$$

Vice versa, given the mean firing frequency λ and the background rate λ_0_, it is

(2) $$\lambda_{1}=\lambda \frac{1}{fT}-\lambda_{0}\left(\frac{1}{fT}-1\right)$$

#### 4.1.2 Non-oscillatory synchronization and rate coding

To investigate firing regimes of non-oscillatory synchronization we can slightly modify the stimulation protocol described above. As before, there are stimulation phases of length *T* where neurons fire at a high rate λ_1_, whereas without stimulation neurons fire at a background rate λ_0_ = 1 spike/s). However, unlike before, the intervals between stimulation events are not fixed. Instead, a stimulation event can start at each time step with probability λ*_e_dt* (independently of previous stimulation events) where we call λ*_e_* the stimulation event rate and *dt* is the simulation step size. Note that different stimulation events may overlap in time. Note that λ*_e_* has the same role as *f* for the oscillatory protocol, and different stimulation events (each of length *T*) may overlap in time. Then the probability that a given time point is not contained within a stimulation event equals the probability that there was no stimulation event starting in the previous *T*/*dt* time steps,

$$\begin{array}{llll} p_{0}: & =\text{pr}\left[\text{no}\;\text{starting}\;\text{event}\;\text{in}\;\text{previous}\;T∕dt\;\text{steps}\right] & & \\ & ={\left(1-\lambda_{e}dt\right)}^{T∕dt} & & \text{(3)} \end{array}$$

and the mean firing rate of a neuron is thus

$$\begin{array}{llll} \lambda & =\frac{\text{pr}\left[\text{spike}\;\text{at}\;\text{simulation}\;\text{step}\;i\right]}{dt}=\frac{\left(1-p_{0}\right)\lambda_{1}dt+p_{0}\lambda_{0}dt}{dt} & & \\ & =\left(1-p_{0}\right)\lambda_{1}+p_{0}\lambda_{0}\;. & & \text{(4)} \end{array}$$

Therefore, for given background rate λ_0_ and given mean firing rate λ the firing rate during the stimulation phases is

(5) $$\lambda_{1}=\frac{\lambda -p_{0}\lambda_{0}}{1-p_{0}}$$

The same stimulation protocol can also be employed to investigate rate coding. Here *T* is typically large (e.g., on the order of several hundred milliseconds), and λ*_e_* much smaller than 1/*T*.

#### 4.1.3 Simplifying assumptions for analyses

Below we conduct two theoretical analyses of the doublet STDP model (see Sections 4.2 and 4.3) and the triplet STDP model (see Section 4.4) where we use a simplified version of the described stimulation protocol.

For the analysis of the doublet STDP model (see Sections 4.2 and 4.3) we consider simple rectangular distributions *G*(Δt) of time lags Δ*t* between presynaptic and postsynaptic spikes (cf., Figures 6A,B). With the exception of rate coding, such distributions are only raw approximations within the Poissonian stimulation framework described above (as are the Gaussians used by Lubenov and Siapas, 2008; cf., Figure 8). Nevertheless, they allow a simple analysis for both linear and non-linear doublet STDP models that is confirmed at least qualitatively by additional simulation experiments (see below).

For the analysis of the triplet STDP model (Section 4.4) we assume an oscillatory stimulation protocol similar as described above, but make three further assumptions (cf., Figure 16): First, both presynaptic and postsynaptic neurons fire exactly once per oscillation period (of length 1/*f*). Second, the firing of the postsynaptic neuron is precisely time-locked to the oscillation, i.e., postsynaptic spikes occur at times *t_i_* = *i*/*f* for *i* = 0, 1,…. Third, firing times of the presynaptic neuron are uniformly distributed around the firing of the postsynaptic neuron within a time interval of length *T*, i.e., presynaptic spikes occur in time intervals *t_i_*∈[*i*/*f* − *T*/2; *i*/*f* + *T*/2].

Due to the simplifications, both analyses lead to closed-form expressions of synaptic weight change (or asymptotic synaptic weights). This allows to overview large parameter ranges to judge whether spike synchronization will lead to either coupling or decoupling of coactivated neurons, and thereby extends the generality of our arguments.

### 4.2 Analysis of doublet STDP

#### 4.2.1 Weight change depends on STDP function F and lag distribution G

For simple doublet STDP models, modification of synaptic weights depends only on the arrival times $t_{s}^{\text{pre}}$ and $t_{s}^{\text{post}}$ of relevant presynaptic and postsynaptic spike pairs (Gerstner et al., 1996; Song et al., 2000; Izhikevich and Desai, 2003; Morrison et al., 2008). Each *relevant* spike pair with time lag $\Delta t:=t_{s}^{\text{post}}-t_{s}^{\text{pre}}$ contributes to weight modification Δ*w* according to an *STDP function* *F*(Δ*t*), for example,

(6) $$F\left(\Delta t\right)=\left\{\begin{array}{cc} A_{+}e^{-\Delta t∕\tau_{+}},\; & \Delta t>0\\ -A_{-}e^{\Delta t∕\tau_{-}},\; & \Delta t<0\\ \; \end{array}\right.,$$

where typically *A*_+_ > *A*_−_ and τ_+_ < τ_−_ such that the integral $\int_{-\infty }^{\infty }\;F\left(t\right)dt$ is negative (Bi and Poo, 1998; Song et al., 2000; Froemke and Dan, 2002; Froemke et al., 2005). All our numerical experiments implementing doublet STDP use parameters from Froemke and Dan (2002): *A*_+_ = 0.0147, *A_−_* = 0.0073, τ_+_ = 13 ms, τ_−_ = 34 ms.

Assuming that the time lags of relevant spike pairs follow a probability distribution with density *G*(Δ*t*), then the expected weight change Δ*w* per pairing is

(7) $$\Delta w=\int_{-\infty }^{\infty }\;F\left(t\right)G\left(t\right)dt\;.$$

A simple example for *G* is a uniform distribution between time lags *t*_1_ and *t*_2_

(8) $$G\left(t\right)=\text{rect}\left(t;c,t_{1},t_{2}\right):=\left\{\begin{array}{cc} c,\; & t_{1}\leq t\leq t_{2}\\ 0,\; & \text{otherwise}\\ \; \end{array}\right.,$$

where *c* is a scaling factor [e.g., *c* = 1/(*t*_2_ − *t*_1_)]. Then the expected weight change is

(9) $$\Delta w=\left\{\begin{array}{cc} cA_{+}\tau_{+}\left(e^{-t_{1}∕\tau_{+}}-e^{-t_{2}∕\tau_{+}}\right)\; & ,\;0\leq t_{1}\leq t_{2}\\ -cA_{-}\tau_{-}\left(e^{t_{2}∕\tau_{-}}-e^{t_{1}∕\tau_{-}}\right)\; & ,\;t_{1}\leq t_{2}\leq 0\\ cA_{+}\tau_{+}\left(1-e^{-t_{2}∕\tau_{+}}\right)\;\\ \;-cA_{-}\tau_{-}\left(1-e^{t_{1}∕\tau_{-}}\right)\; & ,\;t_{1}\leq 0\leq t_{2}\\ \; \end{array}\right.,$$

From this we can compute the expected weight change for various interesting pairing distributions. For example, *all-to-all* (AA) STDP models assume that all spike pairs contribute equally to weight modification (e.g., Gerstner et al., 1996; Song et al., 2000; Knoblauch and Sommer, 2004; Morrison et al., 2008). Then the lag distribution *G* is basically, up to normalization and a time shift *d* due to transmission delays, the *cross correlation function* of the two spike recordings. For example, for independent Poissonian firing *G* is flat and one can use *t*_1_ → −∞ and *t*_2_ → ∞. More interestingly, for occasional epochs of synchronous firing one may choose *t*_1_ = −*T*/2 − *d* and *t*_2_ = *T*/2 − *d* where *T* specifies the width of the synchronization window and *d* specifies an offset corresponding to transmission delays and/or a delay between the firing of the presynaptic and postsynaptic neurons. Then

(10) $$\Delta w=\left\{\begin{array}{cc} cA_{+}\tau_{+}\left(e^{\left(T∕2+d\right)∕\tau_{+}}-e^{\left(-T∕2+d\right)∕\tau_{+}}\right)\; & ,\;d<-T∕2\\ -cA_{-}\tau_{-}\left(e^{\left(T∕2-d\right)∕\tau_{-}}-e^{\left(-T∕2-d\right)∕\tau_{-}}\right)\; & ,\;d>T∕2\\ cA_{+}\tau_{+}\left(1-e^{-\left(T∕2-d\right)∕\tau_{+}}\right)\;\\ \;-cA_{-}\tau_{-}\left(1-e^{-\left(T∕2+d\right)∕\tau_{-}}\right)\; & ,\;|d|\leq T∕2\\ \; \end{array}\right..$$

as used for Figure 6 (with *c* = 1/*T*). Since Δ*w* is linear in *G*, equation (10) can also be used to compute weight changes for cases where *G* is the sum of multiple rectangle functions, for example, a combination of Poissonian background firing, synchronization, and oscillatory components (cf., Figure S1 in Supplementary Material; see also Knoblauch and Hauser, 2011).

### 4.3 Non-linear doublet STDP

#### 4.3.1 Equilibrium weights for power-law doublet STDP

The power-law model of Morrison and colleagues has the same form as equation (6) except that *A*_+_ and *A*_−_ depend on the synaptic weight *w* [cf., Morrison et al., 2007, equation (2.3)]

(11) $$A_{+}=\lambda w_{0}^{1-\mu }w^{\mu }\;\;\text{and}\;\;A_{-}=\lambda \alpha w$$

where reasonable fits to experiments have been obtained for μ = 0.4, *w*_0_ = 1 pA, λ = 0.1, α = 0.11 assuming τ_+_ = τ_−_ = 20 ms. With this we can analyze the effect of coarse synchronization in analogy to equation (10). The following shows that non-linear STDP yields qualitatively similar results as before. First note that *d* < −*T*/2 and *d* > *T*/2 yield unequivocally LTP and LTD, respectively. Notably, in the former case, such models predict unlimited supra-linear LTP during prolonged periods of synchronized activity (whereas LTD is sub-linear). More interesting, for |*d*| ≤ *T*/2, there is an equilibrium weight *w*_∞_ where the expected weight change Δ*w* is zero,

(12) $$\frac{A_{-}}{A_{+}}=\frac{\tau_{+}\left(1-e^{-\left(T∕2-d\right)∕\tau_{+}}\right)}{\tau_{-}\left(1-e^{-\left(T∕2+d\right)∕\tau_{-}}\right)}\;.$$

Thus, solving for *w* yields the equilibrium weight of the power-law model,

(13) $$\frac{w_{\infty }^{\text{power}}}{w_{0}}={\left(\frac{\tau_{+}\left(1-e^{-\left(T/2-d\right)/\tau_{+}}\right)}{\alpha \tau_{-}\left(1-e^{-\left(T/2+d\right)/\tau_{-}}\right)}\right)}^{\frac{1}{1-\mu }}$$

(14) $$\;\underset{\to }{=}{\left(\frac{\tau_{+}}{\alpha \tau_{-}}\right)}^{\frac{1}{1-\mu }}\text{ for }d=\underset{T\to \infty }{0,\;\tau_{+}}=\tau_{-}.$$

Note that in the limit of rate coding and uncorrelated firing (*T* → ∞) all synaptic weights will evolve toward a single value given by equation (14) [which is $w_{\infty }^{\text{power}}\approx 39.6$ for the parameters given below equation (11)]. Similar is true for the particular case of small delays (*d* ≈ 0) and equal time constants for LTP and LTD (τ_+_ = τ_−_). This is consistent with network simulations of Morrison and colleagues that reveal unimodal small-variance distributions of synaptic weights with mean values close to the theoretical value of equation (14)^16^. Surprisingly, the equilibrium synaptic weight *w*_∞_ is independent of firing rate (assuming all-to-all STDP) and, therefore, it seems not reasonable to store information merely by rate coding with uncorrelated firing.

#### 4.3.2 Multiplicative/interpolating doublet STDP

An alternative non-linear STDP model proposed by Gütig et al. (2003) interpolates between additive and multiplicative rules

(15) $$A_{+}=\lambda {\left(1-w\right)}^{\mu }\;\;\text{and}\;\;A_{-}=\lambda \alpha w^{\mu }\;.$$

For example, μ = 1 yields a multiplicative rule (Rubin et al., 2001), whereas μ = 0 yields the additive rule of section 4.2. Here 0 ≤ *w* ≤ 1 for μ > 0. Similarly as before, we obtain the equilibrium weight *w*_∞_ for the interpolating model from equations (10 and 12). For *d* < −*T*/2 and *d* > *T*/2 there is unequivocally LTP and LTD with $w_{\infty }^{\text{inter}}\to 1$ and $w_{\infty }^{\text{inter}}\to 0,$ respectively, whereas

(16) $$w_{\infty }^{\text{inter}}=\frac{1}{\left(1+{\left(\frac{\alpha \tau_{-}\left(1-e^{-\left(T/2+d\right)/\tau_{-}}\right)}{\tau_{+}\left(1-e^{-\left(T/2-d\right)/\tau_{+}}\right)}\right)}^{1/\mu }\right)}$$

(17) $$\;\underset{\to }{=}\frac{1}{1+{\left(\frac{\alpha \tau_{-}}{\tau_{+}}\right)}^{1/\mu }}\text{ for }d=\underset{T\to \infty }{0,\;\tau_{+}}=\tau_{-}$$

is a sigmoid logistic function for |*d*| ≤ T/2. As discussed for the power-law model, all synaptic weights will evolve toward a single value given by equation (17) in the limits of rate coding (*T* → ∞) or small delays and equal time constants (*d* ≈ 0, τ_+_ = τ_−_).

### 4.4 Analysis of triplet STDP

#### 4.4.1 NN-triplet STDP model

For simple doublet STDP models the weight change depends only on the time lags between relevant *pairs* of presynaptic and postsynaptic spikes. It has been argued that these models do not provide good fits to experimental data beyond simple low frequency pairing protocols. To allow meaningful predictions about the outcome of STDP for stimulation protocols including oscillatory and non-oscillatory synchronization with high firing rates, the following considers the triplet model of Pfister and Gerstner (2006). For this model, synaptic weight change depends also on spike triplets in addition to doublets, and it has been shown to fit a much larger set of experimental data including non-linear dependencies on spike rates (Sjöström et al., 2001) as well as triplet and quadruplet experiments (Froemke and Dan, 2002; Wang et al., 2005). For the following analyses and numerical evaluations we consider the nearest-neighbor (NN) variant of triplet STDP described below. Pfister and Gerstner have shown that the NN and all-to-all (AA) variants of triplet (but not doublet) STDP models are basically equivalent in explaining the available experimental data (see Pfister and Gerstner, 2006).

For NN-triplet STDP, each postsynaptic spike at time $t_{\text{post}}^{\left(i\right)}$ leads to synaptic potentiation depending only on the last presynaptic spike time $t_{\text{pre}}^{\left(i\right)}:=\max\left\{t_{\text{pre}}^{\left(j\right)}:t_{\text{pre}}^{\left(j\right)}<t_{\text{post}}^{\left(i\right)}\right\}$ and the last postsynaptic spike time $t_{\text{post}}^{\left(i-1\right)},$

$$\begin{array}{llll} \Delta w & =\exp\left(-\Delta t_{1}∕\tau_{+}\right) & & \\ & \;\times \left(A_{2}^{+}+A_{3}^{+}\exp\left(-\Delta t_{2}∕\tau_{y}\right)\right)\;\text{for each postsynaptic spike} & & \text{(18)} \end{array}$$

where $\Delta t_{1}:=t_{\text{pre}}^{\left(i\right)}-t_{\text{post}}^{\left(i-1\right)}$ and $\Delta t_{2}:=t_{\text{post}}^{\left(i\right)}-t_{\text{pre}}^{\left(i\right)}$ (see Figure 15A). Similarly, each presynaptic spike at time $t_{\text{pre}}^{\left(i\right)}$ induces synaptic depression depending on the last postsynaptic spike time $t_{\text{post}}^{\left(i\right)}:=\max\left\{t_{\text{post}}^{\left(j\right)}:t_{\text{post}}^{\left(j\right)}<t_{\text{pre}}^{\left(i\right)}\right\}$ and the last presynaptic spike time $t_{\text{pre}}^{\left(i-1\right)}$

$$\begin{array}{llll} \Delta w & =-\exp\left(-\Delta t_{1}∕\tau_{-}\right) & & \\ & \;\times \left(A_{2}^{-}+A_{3}^{-}\exp\left(-\Delta t_{2}∕\tau_{x}\right)\right)\;\text{for each presynaptic spike} & & \text{(19)} \end{array}$$

where $\Delta t_{1}:=t_{\text{post}}^{\left(i\right)}-t_{\text{pre}}^{\left(i-1\right)}$ and $\Delta t_{2}:=t_{\text{pre}}^{\left(i\right)}-t_{\text{post}}^{\left(i\right)}$ (see Figure 15B). Note that for zero triplet terms, $A_{3}^{+}=A_{3}^{-}=0,$ the model becomes equivalent to the NN-doublet STDP model (note further that equivalence holds also for stimulation protocols employing very low pairing frequencies such that Δ*t*_2_ ≪ τ*_x_*,τ*_y_*). The following numerical experiments use parameters of Pfister and Gerstner (2006, Tables 3 and 4; minimal parameter sets), fitted to physiological data from hippocampus (Wang et al., 2005), and visual cortex (Sjöström et al., 2001). The hippocampus parameters are $A_{2}^{+}=0.0046,$ $A_{3}^{+}=0.0091,$ $A_{2}^{-}=0.003,$ $A_{3}^{-}=0,$ τ*_x_* = 575 ms, τ*_y_* = 48 ms, τ_+_ = 16.8 ms, τ_−_ = 33.7 ms. The visual cortex parameters are $A_{2}^{+}=0,$ $A_{3}^{+}=0.05,$ $A_{2}^{-}=0.008,$ $A_{3}^{-}=0,$ τ*_x_* = 714 ms, τ*_y_* = 40 ms, τ_+_ = 16.8 ms, τ_−_ = 33.7 ms. For the special case of doublet STDP we used parameters as described above from Froemke and Dan (2002) $\left(A_{2}^{+}=0.0147,\;A_{2}^{-}=0.0073,\;\tau_{+}=13\;\text{ms},\tau_{-}=34\;\text{ms},\;A_{3}^{+}=A_{3}^{-}=0\right).$

In some simulations we scaled these amplitude parameters $\left(A_{2}^{+},A_{2}^{-},A_{3}^{+},A_{3}^{-}\right)$ by a certain factor *s* to have relevant synaptic changes on a time scale comparable to some reference model (e.g., Lubenov and Siapas, 2008; in Figure 4) or to avoid unnecessarily long simulation times^17^. For the triplet STDP simulations of Figures 4 and 5 and Figures S4 and S5 we used scaling factor *s* = 5. In Figure S2 in Supplementary Material we scaled the amplitude parameters of Froemke and Dan (middle and bottom panels) by *s* = 40. For Figure S3 in Supplementary Material we used *s* = 50.

#### 4.4.2 Analysis for oscillatory synchronization

We can compute the expected synaptic change (per presynaptic or postsynaptic spike) from the distribution of time lags Δ*t*_1_ and Δ*t*_2_ (see Figure 15). The following determines such distributions for a stimulation protocol of oscillatory synchronization. For this we make the following assumptions (see Figure 16): (1) Both presynaptic and postsynaptic neurons have oscillatory spike activity with oscillation frequency *f*. (2) Each neuron fires exactly once during each period of the oscillation (i.e., *f* is equal to the neurons’ spike rate λ). (3) The postsynaptic neuron fires without any jitter at times *i*/*f* (*i* = 0,1,2,…). (4) The spike times of the presynaptic neuron are uniformly distributed on the time interval [*i*/*f* *−* *T*/2; *i*/*f* + *T*/2) where *T* ≤ 1/*f* defines the synchronization window. (5) There is an effective transmission delay *d* between presynaptic and postsynaptic neurons with |*d*| < *1*/*f* *−* *T*/2. (6) We finally assume $A_{3}^{-}=0$ as suggested by optimal fits to experimental data (see Tables 3 and 4 in Pfister and Gerstner, 2006).

Let us first consider the distribution of time intervals Δ*t*_1_ and Δ*t*_2_ for each postsynaptic spike corresponding to LTP events [see equation (18) and Figure 15A]. As illustrated by Figure 16, we have to consider the three cases |*d*| < *T*/2, *T*/2 < *d* < 1/*f* *−* *T*/2, and −(1/*f* *−* *T*/2) <*d*< −*T*/2. In any case, it is Δ*t*_2_ = 1/*f* since the interspike interval of the postsynaptic neuron is constant. For |*d*| < *T*/2, with probability (*T*/2 − *d*)/*T* there is a preceding presynaptic spike within the same oscillation period such that Δ*t*_1_ is uniformly distributed in the interval [0;*T*/2 − *d*]. With probability (*T*/2 + *d*)/*T* the preceding presynaptic spike belongs to the preceding oscillation period such that Δ*t*_1_ is uniformly distributed in [1/*f* − *T*/2 − *d*; 1/*f* + *T*/2 − *d*]. For *T*/2 < *d* < 1/*f* *−* *T*/2, it is Δ*t*_1_ ∈ [1/*f* *−* *d* *−* *T*/2; 1/*f* *−* *d* + *T*/2] uniformly. And for −(1/*f* *−* *T*/2) < *d* < −*T*/2, it is Δ*t*_1_ ∈ [−*d* − *T*/2; −*d* + *T*/2] uniformly. Thus, the expected potentiation per postsynaptic spike is

(20) $$\frac{E\left(\Delta w|\text{postsynaptic}\;\text{spike}\right)}{A_{2}^{+}+A_{3}^{+}e^{-1∕\left(f\tau_{y}\right)}}=\left\{\begin{array}{cc} \int_{-d-T∕2}^{-d+T∕2}\;\frac{e^{-t∕\tau_{+}}}{T}dt\; & ,\;-\left(\frac{1}{f}-\frac{T}{2}\right)<d<-\frac{T}{2}\\ \frac{T∕2-d}{T}\int_{0}^{T∕2-d}\;\frac{e^{-t∕\tau_{+}}}{T∕2-d}dt\;\\ \;+\frac{T∕2+d}{T}\int_{1∕f-T∕2-d}^{1∕f+T∕2-d}\;\frac{e^{-t∕\tau_{+}}}{T}dt\; & ,\;|d|<\frac{T}{2}\\ \int_{1∕f-d-T∕2}^{1∕f-d+T∕2}\;\frac{e^{-t∕\tau_{+}}}{T}dt\; & ,\;\frac{T}{2}<d<\frac{1}{f}-\frac{T}{2}\\ \; \end{array}\right.=\left\{\begin{array}{cc} \frac{\tau_{+}\left(e^{-\left(|d|-T∕2\right)∕\tau_{+}}-e^{-\left(|d|+T∕2\right)∕\tau_{+}}\right)}{T}\; & ,\;-\left(\frac{1}{f}-\frac{T}{2}\right)<d<-\frac{T}{2}\\ \frac{\tau_{+}\left(1-e^{-\left(T∕2-d\right)∕\tau_{+}}\right)}{T}\;\\ \;+\frac{\begin{array}{c} \left(T∕2+d\right)\tau_{+}\left(e^{-\left(1∕f-T∕2-d\right)∕\tau_{+}}\right.\\ \;\left.-e^{-\left(1∕f+T∕2-d\right)∕\tau_{+}}\right) \end{array}}{T^{2}}\; & ,\;|d|<\frac{T}{2}\\ \frac{\tau_{+}\left(e^{-\left(1∕f-d-T∕2\right)∕\tau_{+}}-e^{-\left(1∕f-d+T∕2\right)∕\tau_{+}}\right)}{T}\; & ,\;\frac{T}{2}<d<\frac{1}{f}-\frac{T}{2}\\ \; \end{array}\right.$$

We can similarly determine the distribution of time intervals Δ*t*_1_ and Δ*t*_2_ for each presynaptic spike corresponding to LTD events [see equation (19) and Figure 15B]. We have to distinguish between the same three cases as described above (see Figure 16). In any case, we do not explicitly have to compute the distribution of Δ*t*_2_, because of the assumption $A_{3}^{-}=0$. For |*d*| < *T*/2, with probability (*T*/2 + *d*)/*T* there is a preceding postsynaptic spike within the same oscillation period such that Δ*t*_1_ is uniformly distributed in the interval [0;*T*/2 + *d*]. With probability (*T*/2 − *d*)/*T* the preceding postsynaptic spike belongs to the preceding oscillation period such that Δ*t*_1_ is uniformly distributed in [1/*f* − *T*/2 + *d*;1/*f*]. For *T*/2 < *d* < 1/*f* − *T*/2, it is Δ*t*_1_ ∈ [*d* − *T*/2;*d* + *T*/2] uniformly. And for −(1/*f* − *T*/2) < *d* < *T*/2, it is Δ*t*_1_ ∈ [1/*f* + *d* − *T*/2;1/*f* + *d* + *T*/2] uniformly. Thus, the expected potentiation is

(21) $$\frac{E\left(\Delta w|\text{presynaptic}\;\text{spike}\right)}{-A_{2}^{-}}=\left\{\begin{array}{cc} \int_{1∕f+d-T∕2}^{1∕f+d+T∕2}\;\frac{e^{-t∕\tau_{-}}}{T}dt\; & ,\;-\left(\frac{1}{f}-\frac{T}{2}\right)<d<-\frac{T}{2}\\ \frac{T∕2+d}{T}\int_{0}^{T∕2+d}\;\frac{e^{-t∕\tau_{-}}}{T∕2+d}dt\;\\ \;+\frac{T∕2-d}{T}\int_{1∕f-T∕2+d}^{1∕f}\;\frac{e^{-t∕\tau_{-}}}{T∕2-d}dt\; & ,\;|d|<\frac{T}{2}\\ \int_{d-T∕2}^{d+T∕2}\;\frac{e^{-t∕\tau_{-}}}{T}dt\; & ,\;\frac{T}{2}<d<\frac{1}{f}-\frac{T}{2}\\ \; \end{array}\right.=\left\{\begin{array}{cc} \frac{\tau_{-}\left(e^{-\left(1∕f-|d|-T∕2\right)∕\tau_{-}}-e^{-\left(1∕f-|d|+T∕2\right)∕\tau_{-}}\right)}{T}\; & ,\;-\left(\frac{1}{f}-\frac{T}{2}\right)<d<-\frac{T}{2}\\ \frac{\begin{array}{c} \tau_{-}\left(1-e^{-\left(T∕2+d\right)∕\tau_{-}}\right.\\ \;\left.+e^{-\left(1∕f-T∕2+d\right)∕\tau_{-}}-e^{-1∕\left(f\tau_{-}\right)}\right) \end{array}}{T}\; & ,\;|d|<\frac{T}{2}\\ \frac{\tau_{-}\left(e^{-\left(d-T∕2\right)∕\tau_{-}}-e^{-\left(d+T∕2\right)∕\tau_{-}}\right)}{T}\; & ,\;\frac{T}{2}<d<\frac{1}{f}-\frac{T}{2}\\ \; \end{array}\right.$$

per postsynaptic spike. Since we assumed that each neuron fires exactly once per oscillation period, the expected weight change per oscillation period (of length 1/*f*) is thus the sum of equations (20) and (21)

$$\begin{array}{llll} E\left(\Delta w\right) & =E\left(\Delta w|\text{postsynaptic spike}\right) & & \\ & \;+E\left(\Delta w|\text{presynaptic spike}\right). & & \text{(22)} \end{array}$$

We have verified this result by comparison with simulation experiments as documented in a technical report (see Knoblauch and Hauser, 2011, Figure 8). Note that equation (22) applies both to NN-triplet and NN-doublet models.

## Conflict of Interest Statement

The authors declare that the research was conducted in the absence of any commercial or financial relationships that could be construed as a potential conflict of interest.

## Supplementary Material

The Supplementary Material for this article can be found online at http://www.frontiersin.org/Computational_Neuroscience/10.3389/fncom.2012.00055/abstract

## Footnotes

^1^For example, even without considering plasticity of inhibitory neurons, increasing synaptic strength of recurrent excitatory connections can either increase or decrease neuronal synchrony, depending on relative connection strengths between multiple excitatory and inhibitory neuron populations (e.g., for a local circuitry as described by Knoblauch and Palm, 2001 including multiple inhibitory neurons populations; simulation data not shown).

^2^It has been pointed out that this weight dependence is more clearly visible in experimental data for LTD compared to LTP (Bi and Poo, 1998; Morrison et al., 2008).

^3^For oscillatory firing there is one exception to this rule that is possibly relevant for long-range cortico-cortical interactions. As visible in Figure S1C,D in Supplementary Material, AA-STDP can produce strong LTP if the oscillation period is close to the propagation delays (e.g., *d* = 20 ms, *f* = 50 Hz; see also Knoblauch and Sommer, 2003, 2004 for detailed discussions of this effect). However, for local connections under AA-STDP, the weight change (Δ*w* will generally decrease with increasing *f* for any reasonable ranges of *d* and *f*.

^4^It should be noted that NN-doublet STDP models predict depression for very high firing rates when the peak width becomes smaller than the transmission delay *d*.

^5^Note that for the AA-doublet-model increasing firing rate by factor *c* just means to scale the time lag histograms *G*(Δ*t*) of Figure 8 and, thus, also the weight change Δ*w* = ∫ *FG* by the same factor *c*.

^6^The reason for this effect is that lag distributions have stronger central peaks for non-oscillatory compared to oscillatory synchronization (see Figure 8). This is because non-oscillatory stimulation implies a larger variance in the temporal distance between neighboring stimulation periods (see Figure 14) which implies blurring of side peaks and, correspondingly, strengthening of the central peak.

^7^Note that λ/λ_e_ = 2 means bursting with two spikes per stimulation event on average corresponding to a high instantaneous firing rate which should support LTP rather than LTD in the triplet model.

^8^Note that at high firing rates (e.g., λ > 40 Hz for the triplet model with λ/λ_*e*_ = 1) Poissonian firing can more effectively induce LTP than coarsely synchronized regular oscillations. Still, non-oscillatory coarse synchronization induces significantly more LTP than uncorrelated firing even at high rates.

^9^As the NN-doublet model is a special case of the NN-triplet model, this analysis can also be applied to the NN-doublet model for regular oscillations.

^10^It should be noted that LTP for coarse synchronization for the Poissonian protocol does not crucially depend on very small interspike intervals that would be inconsistent with neuronal refractory periods. In fact, such small interspike intervals are extremely seldom for small firing rates and, thus, have only little effect on weight change (data not shown). Consistent with this idea, Figures 4 and 5 proves that triplet STDP fitted to visual cortex data can easily produce LTP even for low firing rates λ ≈ *f* ≈ 4 Hz, regular oscillations, and non-bursting neurons including absolute refractory periods of several milliseconds.

^11^The latter implies that the maximal time lag between presynaptic/postsynaptic spikes (within the same cycle) is actually *T*/2 instead of *T*.

^12^This is so because spike doublets alone are insufficient to evoke LTP due to triplet parameter $A_{2}^{+}=0$. Thus, LTP can be evoked only by a full “LTP triplet” with sufficiently small postsynaptic inter-spike-interval Δ*t*_2_ (see Figure 15, left panel).

^13^Here LTP is restored because bursting decreases effective Δ*t*_2_ of “LTP triplets” (see Figure 15, left panel). Spike bursting is actually necessary only for the triplet STDP model (and other higher-order STDP models where weight change depends only on spike timing configurations of three or more spikes). By contrast, for the voltage-based STDP models, subthreshold synaptic inputs preceding the postsynaptic spike (or tonic subthreshold inputs) can play an equivalent role in promoting LTP at low firing rates.

^14^Our model may also explain why small latency synapses are typically stronger than long latency synapses (cf., Figure 5, lower left panel), and why there is a threshold-like decrease of synaptic efficacy (e.g., around 600 μm distance as reported by Yoshimura et al., 2000). As axons can be much longer than dendrites, the compensation of axonal by dendritic delays in neurons from the same layer (Figure S7A in Supplementary Material) will cease at the latest when *d*_ax_ equals the maximal dendritic delay. At this threshold (which may well correspond to the 600 μm distance reported in the experiments) there should be a drop in the coupling force of synchronization. Minor bidirectional coupling of longer distance neurons may still be possible because *T* increases with distance as explained above.

^15^It is interesting to note that the synaptic connectivity patterns reported for rat visual cortex compared to rat barrel cortex are actually not as different as discussed by Clopath et al. (2010). In fact, Lefort and colleagues suggest that the lower fraction of bidirectional connections in barrel cortex (at chance level) could be due to the larger heterogeneity of cell types involved in their study (Lefort et al., 2009, Table 2) compared to the study of Song et al. (2005) which included only thick tufted layer 5 pyramidal neurons in visual cortex. This seems plausible because there is an earlier study investigating thick tufted layer 5 pyramidal neurons in barrel cortex that reported three times more bidirectional connections than expected from random connectivity (Markram et al., 1997a). This is almost the same result as the factor four dominance reported for visual cortex (Song et al., 2005). Thus, the differences in reported connectivity patterns may well be a result of different experimental selection protocols rather than different coding principles. As long as connectivity analyses do not include individual synaptic strengths, remaining differences in connectivity patterns may also attributable to differences in the dynamics of structural plasticity (Holtmaat and Svoboda, 2009; Knoblauch, 2009; Knoblauch et al., 2010; Fu and Zuo, 2011), i.e., the seemingly random connectivity pattern reported by Lefort et al. (2009) could also be explained by a lower degree of structural plasticity in certain cortical areas, e.g., without any structural plasticity the connectivity pattern remains fixed and random from the beginning and, in particular, does not depend on the neural code.

^16^For example, simulation experiments of Morrison et al. (2007, Figure 2) yield *w*_∞_ = 45.5 ± 4.0 pA, whereas equation (14) yields $w_{\infty }^{\text{power}}=44.83\;\text{pA}$ (for that particular simulation experiment, Morrison and colleagues used α = 0.1201 instead of α = 0.11). Interestingly, Morrison and colleagues report a dependence on firing rates, whereas our theory suggests (for all-to-all doublet STDP) that the equilibrium weight is independent of firing rate (and, thus, it seems not a good idea to use firing rates as a means to store information in synaptic weights). Our interpretation is that the dependence on firing rate observed by Morrison and colleagues is rather a transient due to spike correlations induced by switching on input currents during simulations to modify firing rates (whereas weights should actually return to the equilibrium value for sufficiently long simulation times).

^17^Note that, for linear STDP models, scaling of amplitudes is equivalent to increasing simulation time.

## References

[B1] Abeles M. (1982). Local Cortical Circuits. Berlin: Springer

[B2] Attwell D. Laughlin S. (2001). An energy budget for signaling in the grey matter of the brain. J. Cereb. Blood Flow Metab. 21, 1133–1145 11598490 10.1097/00004647-200110000-00001

[B3] Benabid A. Chabardes S. Mitrofanis J. Pollak P. (2009). Deep brain stimulation of the subthalamic nucleus for the treatment of parkinson’s disease. Lancet Neurol. 8, 67–81 10.1016/S1474-4422(08)70291-6 19081516

[B4] Bi G. (2002). Spatiotemporal specificity of synaptic plasticity: cellular rules and mechanisms. Biol. Cybern. 87, 319–332 10.1007/s00422-002-0349-7 12461623

[B5] Bi G. Poo M. (1998). Synaptic modifications in cultured hippocampal neurons: dependence on spike timing, synaptic strength, and postsynaptic cell type. J. Neurosci. 18, 10464–10472 9852584 10.1523/JNEUROSCI.18-24-10464.1998 PMC6793365

[B6] Bi G. Wang H. (2002). Temporal asymmetry in spike timing-dependent synaptic plasticity. Physiol. Behav. 77, 551–555 10.1016/S0031-9384(02)00933-2 12526998

[B7] Bienenstock E. Cooper L. Munro P. (1982). Theory for the development of neuron selectivity: orientation specificity and binocular interaction in visual cortex. J. Neurosci. 2, 32–48 7054394 10.1523/JNEUROSCI.02-01-00032.1982 PMC6564292

[B8] Braitenberg V. (1978). “Cell assemblies in the cerebral cortex,” in Lecture Notes in Biomathematics (21). Theoretical Approaches to Complex Systems, eds Heim R. Palm G. (Berlin: Springer-Verlag), 171–188

[B9] Brunel N. (2000). Dynamics of sparsely connected networks of excitatory and inhibitory spiking neurons. J. Comput. Neurosci. 8, 183–208 10.1023/A:1008925309027 10809012

[B10] Burkitt A. Meffin M. Grayden D. (2004). Spike-timing-dependent plasticity: the relationship to rate-based learning for models with weight dynamics determined by a stable fixed point. Neural Comput. 16, 885–940 10.1162/089976604773135041 15070504

[B11] Buzsaki G. (2006). Rhythms of the Brain. New York: Oxford University Press

[B12] Buzsaki G. (2010). Neural syntax: cell assemblies, synapsembles, and readers. Neuron 64, 362–385 10.1016/j.neuron.2010.09.023 21040841 PMC3005627

[B13] Clopath C. Büsing L. Vasilaki E. Gerstner W. (2010). Connectivity reflects coding: a model of voltage-based STDP with homeostasis. Nat. Neurosci. 13, 344–352 10.1038/nn.2479 20098420

[B14] Dan Y. Poo M.-M. (2006). Spike timing-dependent plasticity: from synapse to perception. Physiol. Rev. 86, 1033–1048 10.1152/physrev.00030.2005 16816145

[B15] DeWeese M. Zador A. (2006). Non-gaussian membrane potential dynamics imply sparse, synchronous activity in auditory cortex. J. Neurosci. 26, 12206–12218 10.1523/JNEUROSCI.2813-06.2006 17122045 PMC6675435

[B16] Diesmann M. Gewaltig M. Aertsen A. (1999). Stable propagation of synchronous spiking in cortical neural networks. Nature 402, 529–533 10.1038/990101 10591212

[B17] Eckhorn R. Bruns A. Saam M. Gail A. Gabriel A. Brinksmeyer H. (2001). Flexible cortical gamma-band correlations suggest neural principles of visual processing. Vis. cogn. 8, 519–530 10.1080/13506280143000098

[B18] Fell J. Axmacher N. (2011). The role of phase synchronization in memory processes. Nat. Rev. Neurosci. 12, 105–118 10.1038/nrn2979 21248789

[B19] Fries P. Reynolds J. Rorie A. Desimone R. (2001). Modulation of oscillatory neuronal synchronization by selective visual attention. Science 291, 1560–1563 10.1126/science.1055465 11222864

[B20] Froemke R. Dan Y. (2002). Spike-timing-dependent synaptic modification induced by natural spike trains. Nature 416, 433–438 10.1038/416433a 11919633

[B21] Froemke R. Poo M. Dan Y. (2005). Spike-timing-dependent synaptic plasticity depends on dendritic location. Nature 434, 221–225 10.1038/nature03366 15759002

[B22] Fu M. Zuo Y. (2011). Experience-dependent structural plasticity in the cortex. Trends Neurosci. 34, 177–187 10.1016/j.tins.2011.02.001 21397343 PMC3073830

[B23] Gerstner W. Kempter R. van Hemmen J. Wagner H. (1996). A neuronal learning rule for sub-millisecond temporal coding. Nature 386, 76–78 10.1038/383076a0 8779718

[B24] Girard P. Hupe J. Bullier J. (2001). Feedforward and feedback connections between areas V1 and V2 of the monkey have similar rapid conduction velocities. J. Neurophysiol. 85, 1328–1331 11248002 10.1152/jn.2001.85.3.1328

[B25] Griffith J. (1963). On the stability of brain structures. Biophys. J. 3, 299–308 10.1016/S0006-3495(63)86822-8 13950414 PMC1366448

[B26] Gütig R. Aharonov R. Rotter S. Sompolinsky H. (2003). Learning input correlations through non-linear temporally asymmetric Hebbian plasticity. J. Neurosci. 23, 3697–3714 12736341 10.1523/JNEUROSCI.23-09-03697.2003 PMC6742165

[B27] Hebb D. (1949). The Organization of Behavior. A Neuropsychological Theory. New York: Wiley

[B28] Holtmaat A. Svoboda K. (2009). Experience-dependent structural synaptic plasticity in the mammalian brain. Nat. Rev. Neurosci. 10, 647–658 10.1038/nrn2721 19693029

[B29] Hopfield J. (1982). Neural networks and physical systems with emergent collective computational abilities. Proc. Natl. Acad. Sci. U.S.A. 79, 2554–2558 10.1073/pnas.79.8.2554 6953413 PMC346238

[B30] Izhikevich E. Desai N. (2003). Relating STDP to BCM. Neural Comput. 15, 1511–1523 10.1162/089976603321891783 12816564

[B31] Jadhav S. Wolfe J. Feldman D. (2009). Sparse temporal coding of elementary tactile features during active whisker sensation. Nat. Neurosci. 12, 792–800 10.1038/nn.2328 19430473

[B32] Jutras M. Buffalo E. (2010). Synchronous neural activity and memory formation. Curr. Opin. Neurobiol. 20, 150–155 10.1016/j.conb.2010.02.006 20303255 PMC2862842

[B33] Kampa B. Stuart G. (2006). Calcium spikes in basal dendrites of layer 5 pyramidal neurons during action potential bursts. J. Neurosci. 26, 7424–7432 10.1523/JNEUROSCI.3062-05.2006 16837590 PMC6674200

[B34] Knoblauch A. (2009). “The role of structural plasticity and synaptic consolidation for memory and amnesia in a model of cortico-hippocampal interplay,” in Connectionist Models of Behavior and Cognition II: Proceedings of the 11th Neural Computation and Psychology Workshop, eds Mayor J. Ruh N. Plunkett K. (Singapore: World Scientific Publishing), 79–90

[B35] Knoblauch A. (2011). Neural associative memory with optimal bayesian learning. Neural Comput. 23, 1393–1451 10.1162/NECO_a_00127 21395440

[B36] Knoblauch A. Hauser F. (2011). Stdp, Temporal Coding, and Anatomical Connectivity Patterns. HRI-EU Report 11-36. Offenbach/Main: Honda Research Institute Europe GmbH

[B37] Knoblauch A. Palm G. (2001). Pattern separation and synchronization in spiking associative memories and visual areas. Neural Netw. 14, 763–780 10.1016/S0893-6080(01)00084-3 11665769

[B38] Knoblauch A. Palm G. (2002a). Scene segmentation by spike synchronization in reciprocally connected visual areas. II. Global assemblies and synchronization on larger space and time scales. Biol. Cybern. 87, 168–184 10.1007/s00422-002-0332-3 12200613

[B39] Knoblauch A. Palm G. (2002b). Scene segmentation by spike synchronization in reciprocally connected visual areas. I. Local effects of cortical feedback. Biol. Cybern. 87, 151–167 10.1007/s00422-002-0332-3 12200612

[B40] Knoblauch A. Palm G. Sommer F. (2010). Memory capacities for synaptic and structural plasticity. Neural Comput. 22, 289–341 10.1162/neco.2009.08-07-588 19925281

[B41] Knoblauch A. Sommer F. (2003). Synaptic plasticity, conduction delays, and inter-areal phase relations of spike activity in a model of reciprocally connected areas. Neurocomputing 52–54, 301–306. 10.1016/S0925-2312(02)00792-0

[B42] Knoblauch A. Sommer F. (2004). Spike-timing-dependent synaptic plasticity can form “zero lag” links for cortical oscillations. Neurocomputing 58–60, 185–190. 10.1016/j.neucom.2004.01.041

[B43] Kozloski J. Cecchi G. (2008). Topological Effects of Synaptic Spike Timing-Dependent Plasticity. Available at http://arxiv.org/abs/0810.0029

[B44] Lamsa K. Kullmann D. Woodin M. (2010). Spike-timing dependent plasticity in inhibitory circuits. Front. Synaptic Neurosci. 2, 1–8 21423494 10.3389/fnsyn.2010.00008 PMC3059674

[B45] Lansner A. (2009). Associative memory models: from the cell-assembly theory to biophysically detailed cortex simulations. Trends Neurosci. 32, 178–186 10.1016/j.tins.2008.12.002 19187979

[B46] Laughlin S. Sejnowski T. (2003). Communication in neuronal networks. Science 301, 1870–1874 10.1126/science.1089662 14512617 PMC2930149

[B47] Lefort S. Tomm C. Sarria J. Petersen C. (2009). The excitatory neuronal network of the C2 barrel column in mouse primary somatosensory cortex. Neuron 61, 301–316 10.1016/j.neuron.2008.12.020 19186171

[B48] Lennie P. (2003). The cost of cortical computation. Curr. Biol. 13, 493–497 10.1016/S0960-9822(03)00135-0 12646132

[B49] Levy N. Horn D. Meilijson I. Ruppin E. (2001). Distributed synchrony in a cell assembly of spiking neurons. Neural Netw. 14, 815–824 10.1016/S0893-6080(01)00044-2 11665773

[B50] Liao D. Jones A. Malinow R. (1992). Direct measurement of quantal changes underlying long-term potentiation in ca1 hippocampus. Neuron 9, 1089–1097 10.1016/0896-6273(92)90068-O 1334418

[B51] Lu J. Li C. Zhao J. Poo M. Zhang X. (2007). Spike-timing-dependent plasticity of neocortical excitatory synapses on inhibitory interneurons depends on target cell type. J. Neurosci. 27, 9711–9720 10.1523/JNEUROSCI.4871-06.2007 17804631 PMC6672961

[B52] Lubenov E. Siapas A. (2008). Decoupling through synchrony in neuronal circuits with propagation delays. Neuron 58, 118–131 10.1016/j.neuron.2008.01.036 18400168

[B53] Markram H. Lübke J. Frotscher M. Roth A. Sakmann B. (1997a). Physiology and anatomy of synaptic connections between thick tufted pyramidal neurones in the developing rat neocortex. J. Physiol. 500(Pt 2), 409–440 9147328 10.1113/jphysiol.1997.sp022031 PMC1159394

[B54] Markram H. Lübke J. Frotscher M. Sakmann B. (1997b). Regulation of synaptic efficacy by coincidence of postsynaptic APs and EPSPs. Science 275, 213–215 10.1126/science.275.5297.213 8985014

[B55] Markram H. Toledo-Rodriguez M. Wang Y. Gupta A. Silberberg G. Wu C. (2004). Interneurons of the neocortical inhibitory system. Nat. Rev. Neurosci. 5, 793–807 10.1038/nrn1519 15378039

[B56] Marr D. (1971). Simple memory: a theory for archicortex. Philos. Trans. R. Soc. Lond. B Biol. Sci. 262, 24–81 10.1098/rstb.1971.0078 4399412

[B57] Miltner W. Braun C. Arnold M. Witte H. Taub E. (1999). Coherence of gamma-band EEG activity as a basis for associative learning. Nature 397, 434–436 10.1038/17126 9989409

[B58] Montgomery J. Madison D. (2004). Discrete synaptic states define a major mechanism of synapse plasticity. Trends Neurosci. 27, 744–750 10.1016/j.tins.2004.10.006 15541515

[B59] Montgomery J. Pavlidis P. Madison D. (2001). Pair recordings reveal all-silent synaptic connections and the postsynaptic expression of long-term potentiation. Neuron 29, 691–701 10.1016/S0896-6273(01)00244-6 11301028

[B60] Morrison A. Aertsen A. Diesmann M. (2007). Spike-timing-dependent plasticity in balanced random networks. Neural Comput. 19, 1437–1467 10.1162/neco.2007.19.1.47 17444756

[B61] Morrison A. Diesmann M. Gerstner W. (2008). Phenomenological models of synaptic plasticity based on spike timing. Biol. Cybern. 98, 459–478 10.1007/s00422-008-0233-1 18491160 PMC2799003

[B62] O’Connor D. Wittenberg G. Wang S.-H. (2005). Graded bidirectional synaptic plasticity is composed of switch-like unitary events. Proc. Natl. Acad. Sci. U.S.A. 102, 9679–9684 10.1073/pnas.0502332102 15983385 PMC1172253

[B63] Palm G. (1980). On associative memories. Biol. Cybern. 36, 19–31 10.1007/BF00337019 7353062

[B64] Palm G. (1982). Neural Assemblies. An Alternative Approach to Artificial Intelligence. Berlin: Springer

[B65] Petersen C. Malenka R. Nicoll R. Hopfield J. (1998). All-or-none potentiation at CA3-CA1 synapses. Proc. Natl. Acad. Sci. U.S.A. 95, 4732–4737 10.1073/pnas.95.1.310 9539807 PMC22559

[B66] Pfister J.-P. Gerstner W. (2006). Triplets of spikes in a model of spike timing-dependent plasticity. J. Neurosci. 26, 9673–9682 10.1523/JNEUROSCI.1425-06.2006 16988038 PMC6674434

[B67] Pfister J.-P. Tass P. (2010). STDP in oscillatory recurrent networks: theoretical conditions for desychronization and applications to deep brain stimulation. Front. Comput. Neurosci. 4:22 10.3389/fncom.2010.00022 20802859 PMC2928668

[B68] Pulvermüller F. (2003). The Neuroscience of Language: On Brain Circuits of Words and Serial Order. Cambridge: Cambridge University Press

[B69] Rubin J. Lee D. Sompolinsky H. (2001). Equilibrium properties of temporally asymmetric Hebbian plasticity. Phys. Rev. Lett. 86, 364–367 10.1103/PhysRevLett.86.364 11177832

[B70] Shadlen M. Movshon J. (1999). Synchrony unbound: a critical evaluation of the temporal binding hypothesis. Neuron 24, 67–77 10.1016/S0896-6273(00)80822-3 10677027

[B71] Shadlen M. Newsome W. (1994). Noise, neural codes and cortical organization. Curr. Opin. Neurobiol. 4, 569–579 10.1016/0959-4388(94)90059-0 7812147

[B72] Singer W. Gray C. (1995). Visual feature integration and the temporal correlation hypothesis. Annu. Rev. Neurosci. 18, 555–586 10.1146/annurev.ne.18.030195.003011 7605074

[B73] Sjöström P. Turrigiano G. Nelson S. (2001). Rate, timing, and cooperativity jointly determine cortical synaptic plasticity. Neuron 32, 1149–1164 10.1016/S0896-6273(01)00542-6 11754844

[B74] Song S. Abbott L. (2001). Cortical development and remapping through spike timing-dependent plasticity. Neuron 32, 339–350 10.1016/S0896-6273(01)00451-2 11684002

[B75] Song S. Miller K. Abbott L. (2000). Competitive Hebbian learning through spike-timing-dependent synaptic plasticity. Nat. Neurosci. 3, 919–926 10.1038/78829 10966623

[B76] Song S. Sjöström P. Reigl M. Nelson S. Chklovskii D. (2005). Highly non-random features of synaptic connectivity in local cortical circuits. PLoS Biol. 3, e68 10.1371/journal.pbio.0030068 15737062 PMC1054880

[B77] Swadlow H. (2000). “Information flow along neocortical axons,” in Time and the Brain, Conceptual Advances in Brain Research, Chap. 4, ed. Miller R. (Amsterdam: Harwood Academic Publishers), 131–155

[B78] Theunissen F. Miller J. (1995). Temporal encoding in nervous systems: a rigorous definition. J. Comput. Neurosci. 2, 149–162 10.1007/BF00961885 8521284

[B79] van Rossum M. Bi G. Turrigiano G. (2000). Stable Hebbian learning from spike-timing-dependent plasticity. J. Neurosci. 20, 8812–8821 11102489 10.1523/JNEUROSCI.20-23-08812.2000 PMC6773092

[B80] VanRullen R. Guyonneau R. Thorpe S. (2005). Spike times make sense. Trends Neurosci. 28, 1–4 10.1016/j.tins.2004.10.010 15626490

[B81] Vogels T. Rajan K. Abbott L. (2005). Neural network dynamics. Annu. Rev. Neurosci. 28, 357–376 10.1146/annurev.neuro.28.061604.135637 16022600

[B82] Vogels T. Sprekeler H. Zenke F. Clopath C. Gerstner W. (2011). Inhibitory plasticity balances excitation and inhibition in sensory pathways and memory networks. Science 334, 1569–1573 10.1126/science.1211095 22075724

[B83] Wang H. Gerkin R. Nauen D. Bi G. (2005). Coactivation and timing-dependent integration of synaptic potentiation and depression. Nat. Neurosci. 8, 187–193 10.1038/nn1387 15657596

[B84] Yen S. Baker J. Gray C. (2007). Heterogeneity in the responses of adjacent neurons to natural stimuli in cat striate cortex. J. Neurophysiol. 97, 1326–1341 10.1152/jn.00747.2006 17079343

[B85] Yoshimura Y. Sato H. Imamura K. Watanabe Y. (2000). Properties of horizontal and vertical inputs to pyramidal cells in the superficial layers of the cat visual cortex. J. Neurosci. 20, 1931–1940 10684894 10.1523/JNEUROSCI.20-05-01931.2000 PMC6772908

